# Low Dark Current Organic Photodetectors Utilizing Highly Cyanated Non-fullerene Acceptors

**DOI:** 10.1021/acsami.2c10197

**Published:** 2022-08-16

**Authors:** Panagiota Kafourou, Zhuoran Qiao, Máté Tóth, Filip Aniés, Flurin Eisner, Nicola Gasparini, Martin Heeney

**Affiliations:** † Department of Chemistry and Centre for Processable Electronics, Imperial College London, London, W12 0BZ, UK; ‡ Department of Physics, and Centre for Processable Electronics, Imperial College London, London, SW7 2AZ, UK

**Keywords:** organic photodetector, non-fullerene acceptor, organic semiconductor, bulk heterojunction, cyanation, dark current

## Abstract

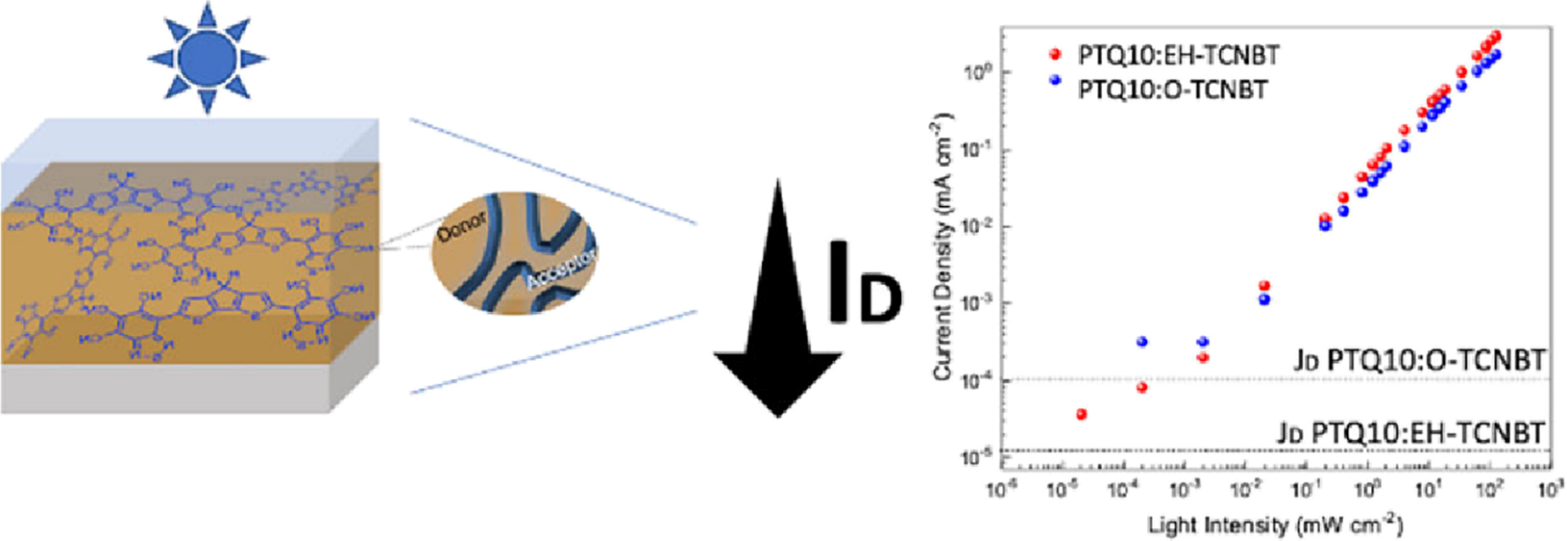

Organic materials combining high electron affinity with strong absorption in the visible spectrum are of interest for photodetector applications. In this study, we report two such molecular semiconductors, based upon an acceptor–donor–acceptor (A-D-A) approach. Coupling of an acceptor end group, 2,1,3-benzothiadiazole-4,5,6-tricarbonitrile (TCNBT), with a donor cyclopentadithiophene core affords materials with a band gap of 1.5 eV and low-lying LUMO levels around −4.2 eV. Both materials were readily synthesized by a one-pot nucleophilic displacement of a fluorinated precursor by cyanide. The two acceptors only differ in the nature of the solubilizing alkyl chain, which is either branched 2-ethyl hexyl (EH-TCNBT) or linear octyl (O-TCNBT). Both acceptors were blended with polymer donor PTQ10 as an active layer in OPDs. Significant device differences were observed depending on the alkyl chain, with the branched acceptor giving the optimum performance. Both acceptors exhibited very low dark current densities, with values up to 10^–5^ mA cm^–2^ at −2 V, highlighting the potential of the highly cyanated cores (TCNBT) as acceptor materials.

## Introduction

Organic semiconductors (OSCs) are of great importance due to their diverse and wide-ranging applications, from optical communication to biomedical imaging.^1,2^ Compared to their inorganic counterparts, they typically offer mechanical flexibility, structural tunability, and the potential for large-area manufacturing.^3^ After the success of organic materials in light emitting diodes (OLEDs) and organic photovoltaics (OPVs), another emerging technology, that of organic photodetectors (OPDs), is gaining interest, partly due to the similarities between OPDs and OPVs. The performance of OPDs is characterized by several parameters; noise equivalent power (NEP), which is related to responsivity (*R*) and noise current (*i*_n_), dark current (*J*_d_), signal to noise ratio (SNR), specific detectivity (D*), transient times, cutoff frequency, and linear dynamic range.^4^ The simultaneous optimization of multiple parameters is a challenging requirement to optimize OPD performance. Additionally, the conversion of low levels of light into detectable electric signals often requires a large negative operating voltage, making current OPD devices inefficient. Many OPD devices are based on solution processed π-conjugated systems and, via careful selection of materials, can achieve a good response in the UV, visible, and near-IR regions.^5^ Despite the importance of the active layer, device engineering and optimization are also crucial for high-performance photodetectors, where active layer thickness, interlayer materials, and electrodes are significant.

The bulk-heterojunction (BHJ) OPD architecture, which has also been widely used in OPVs, is an efficient approach to tune light harvesting and improve free charge generation.^6^ A BHJ is formed by blending electron-donating with electron-accepting materials. The resulting heterojunction gives rise to an energy offset greater than the exciton binding energy, facilitating charge generation following photon absorption. For detection of high energy photons of UV–visible light, broad-band OPDs based on phenyl-C_61_-butyric acid methyl ester (PCBM) have been previously fabricated.^7−9^ The absence of a spectral response in the longwave region limits PCBM and its analogues in full-visible range photodetection.

On the other hand, non-fullerene acceptors, with high absorption coefficients, tunable optoelectronic properties, and low rates of charge recombination are appealing for photodetector applications.^10^ Various reported NFAs have been implemented in OPD devices including ITIC,^11^ O-IDTBR,^12,13^ and O-FBR.^12^ ITIC has been utilized in ternary blends in P3HT:PC_71_BM:ITIC-based OPD devices with specific detectivity beyond 10^12^ Jones in the visible region ranging from 380 to 760 nm and responsivity of 0.25 A W^–1^ at 710 nm.^11^ When O-IDTBR was blended with P3HT, P3HT:O-IDTBR-based OPD exhibited dark current *J*_d_ ≈ 30 nA cm^–2^ and responsivity of 0.42 A W^–1^ at 755 nm,^13^ whereas PTQ10:O-IDTBR-based OPDs exhibited dark current *J*_d_ ≈ 0.84 nA cm^–2^ at 560 nm and responsivity of 0.003 AW^–1^.^12^ O-FBR was used in PTQ10:O-FBR-based OPDs and exhibited a dark current of 0.17 nA cm^–2^ at 610 nm and responsivity of 0.34 AW^–1^.^12^ However, there is still room for considerable improvement in the performance of current blends, especially in the near-infrared region.

In our search for new electron accepting materials for OPD applications, we were interested in the application of the strong electron acceptor, 2,1,3-benzo-thiadiazole-4,5,6-tricarbonitrile, TCNBT, as an end group for acceptor–donor–acceptor (A-D-A) structures.^14^ Despite the promising performance of TCNBT as an electron transporting material in transistor applications, its performance as an acceptor in OPD/OPV blends has not been investigated to the best of our knowledge. We have recently demonstrated that TCNBT can be used to afford a very low band gap (*ca*. 1 eV) n-type semiconductor, when used in combination with an extended electron rich core.^15^ Here, in order to promote visible light absorption, we combined TCNBT with a less electron-rich cyclopentadithiophene (CDT)^16^ core in order to form a wider band gap material. Side chains are known to play a critical role in influencing solubility and the ability of the material to aggregate and crystallize, as well as affecting blend stability and microstructures.^17−19^ Thus, we studied the nature of the sidechain on the CDT core, investigating a branched side chain, 2-ethyl hexyl,^18,20,21^ in comparison to a linear, *n*-octyl sidechain. Both materials were synthesized from their fluorinated precursors **EH-TFBT** and **O-TFBT**, via six-fold aromatic nucleophilic substitution with cyanide. Both cyanated materials demonstrated a band gap of 1.5 eV in the solid state, and their optoelectronic properties were compared and contrasted with their fluorinated counterparts. Blends of **EH-TCNBT** and **O-TCNBT** with PTQ10 were optimized in OPD devices. Promising device performance was obtained, with minimal dark current densities of 10^–4^ to 10^–5^ mA cm^–2^. The PTQ10:**EH-TCNBT**-based OPDs showed the best performance, with specific detectivity (D*) measured at 2.86 × 10^11^ Jones at 750 nm and dark current at 1.27 × 10^–5^ mA cm^–2^ at an applied bias of −2 V. On the other hand, OPDs with the linear octyl chain acceptor showed higher D* and dark current values at 2.09 × 10^9^ Jones at 700 nm and 1.01 × 10^–4^ mA cm^–2^ at applied bias at −2 V, respectively. These results demonstrate that molecular design and small changes in the molecular backbone are useful tools for optimizing the active layer of an OPD device.

## Synthesis and Characterization

**Scheme 1:**
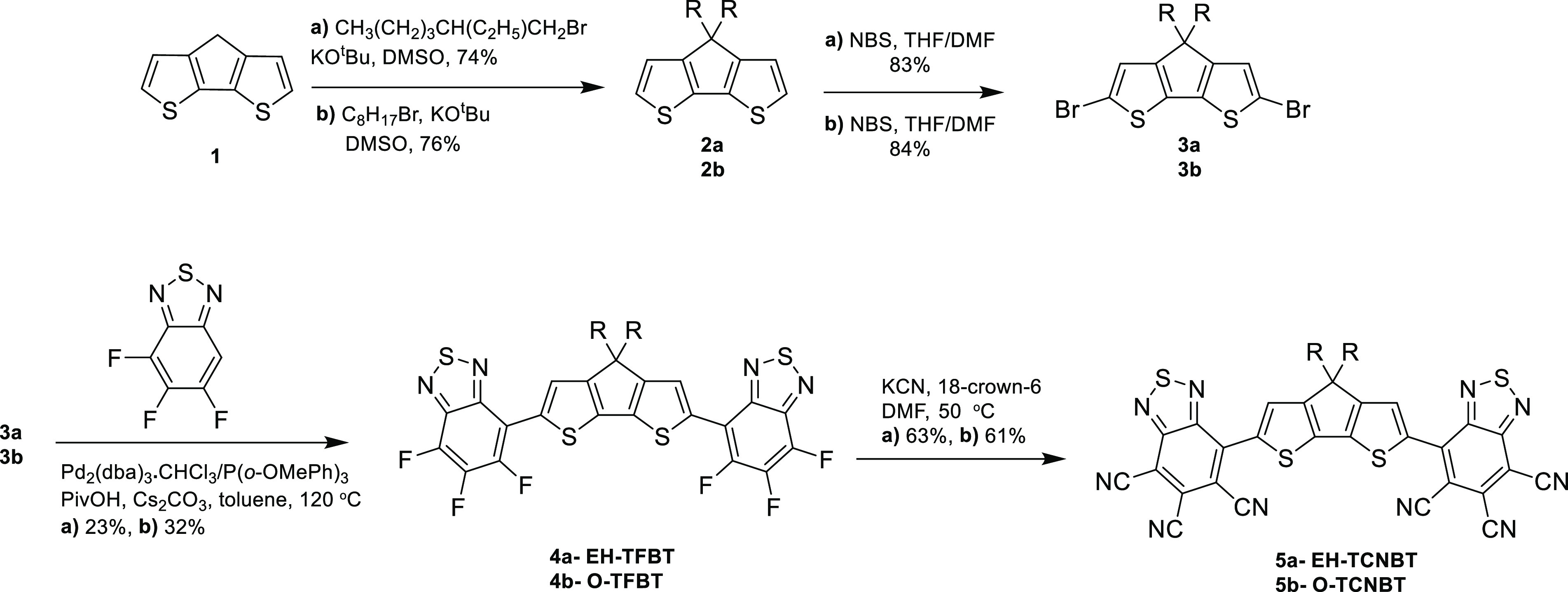
Synthesis of Fluorinated Precursors **EH-TFBT** and **O-TFBT** and Cyanated **EH-TCNBT** and **O-TCNBT**

The synthesis of **EH-TCNBT** and **O-TCNBT** is shown in Scheme 1. Commercially available cyclopentadithiophene **1** was alkylated with *n*-octyl bromide or 1-bromo-2-ethylhexane, by rection with potassium *tert*-butoxide in DMSO, resulting in **2a** and **2b**, respectively. Bromination with NBS in a mixture of THF/DMF produced intermediates **3a** and **3b** that were subsequently coupled with trifluorobenzothiadiazole (TFBT) under direct arylation conditions, yielding intermediates **EH-TFBT** and **O-TFBT**, respectively.^14,15^ It should be noted that product **3a** (and **EH-TFBT**) is formed as a mixture of stereoisomers due to the use of racemic 1-bromo-2-ethylhexane. This is clearly observed in the ^1^H NMR spectra of **3a**, in which the aromatic signals are present as an apparent triplet due to the presence of different diastereomers, versus a singlet for **3b** with linear octyl sidechains (Figures S14 and S18, respectively). **EH-TFBT** and **O-TFBT** were isolated in moderate 20–38% yields, depending on the scale of the reaction. Finally, both **EH-TFBT** and **O-TFBT** were treated with potassium cyanide and 18-crown-6 in the presence of DMF. Direct displacement of all fluorines with cyanide via 6-fold nucleophilic substitution was successful, resulting in **EH-TCNBT** and **O-TCNBT** in good yields, 63% and 78%, respectively. Fluorinated and cyanated compounds were highly soluble at room temperature in common chlorinated organic solvents like chloroform, and their structures were confirmed by a combination of NMR and mass spectroscopy (see Supporting Information).

**Figure 1:**
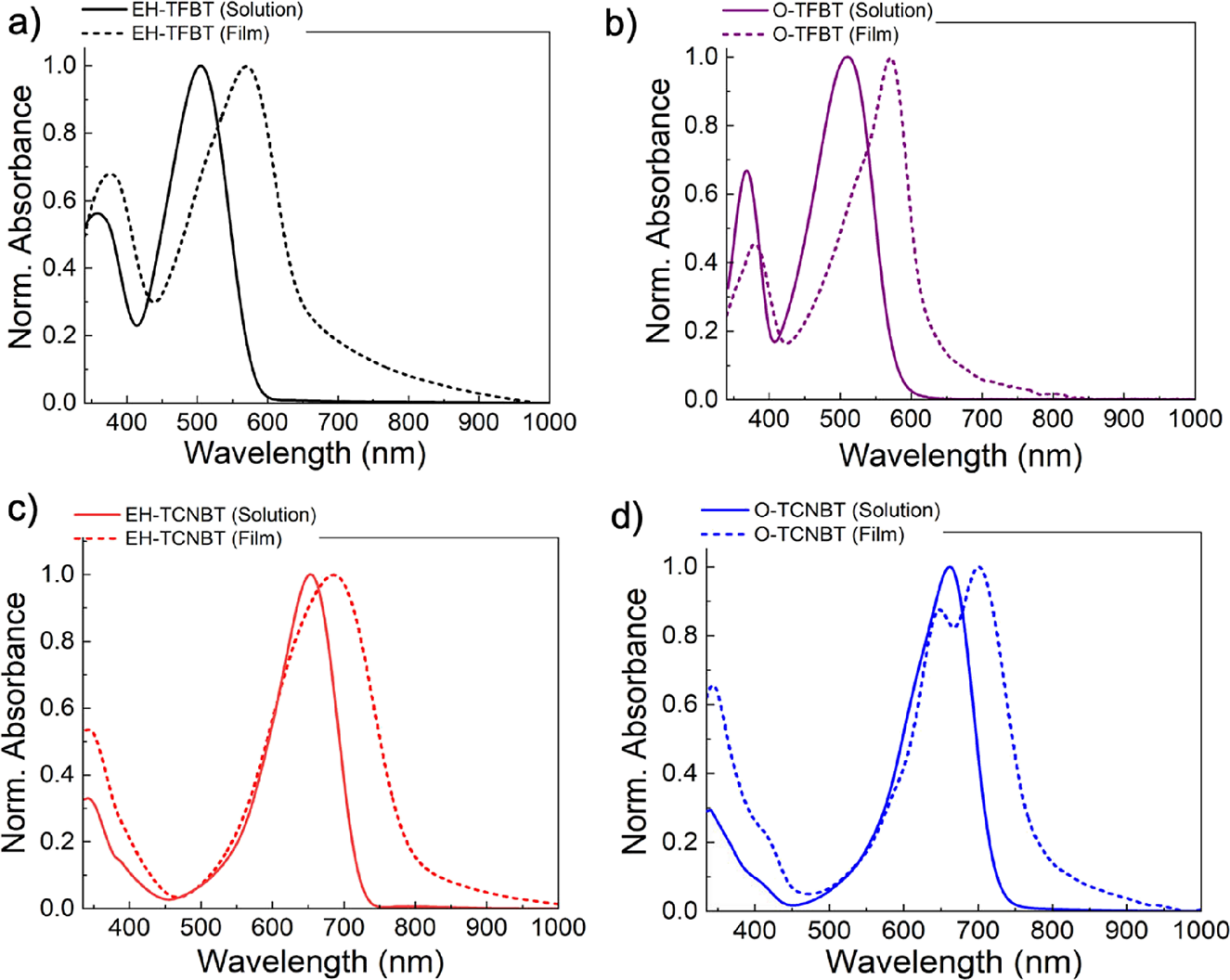
UV–Vis absorption spectra of (a) **EH-TFBT** and (b) **O-TFBT** in chloroform solution and at 80 °C annealed films, (c) **EH-TCNBT**, and (d) **O-TCNBT** solid state absorption as a function of temperature.

The UV–Vis absorption spectra of fluorinated precursors **EH-TFBT** and **O-TFBT** are shown in Figure 1a,b, respectively. The solution spectra were recorded in chloroform, and the films were spin-coated from chloroform solution and annealed at 80 °C. **EH-TFBT** shows two peaks at 359 and 506 nm in solution that are red shifted by 19 nm and 48 nm in the solid state. The optical band gap was estimated by the onset of the absorption spectra at 2.1 and 1.8 eV in solution and solid state, respectively. Substituting a branched solubilizing chain with a linear alkyl chain resulted in minor alterations in the optical properties of the materials, with **O-TFBT** exhibiting a red shift of the absorption peaks in solution, to 368 and 510 nm. These are further red-shifted by 12 and 61 nm, respectively, upon moving to the solid state. The intensity of the high energy peak relative to the lower energy is reduced in the solid state in the case of **O-TFBT**, whereas the opposite happens in the case of **EH-TFBT**. Additionally, a more pronounced vibronic shoulder around 520 nm is apparent in the case of **O-TFBT**, whereas **EH-TFBT** is broader in the sold state. The optical band gap was estimated by the onset of the absorption spectra, at 2.1 and 1.8 eV in solution and solid state, respectively. It is worth noting that annealing has a different effect in both materials. In the case of **EH-TFBT** (Figure S1a), annealing at 80 °C causes a redshift, which gradually blueshifts upon higher annealing temperatures. **O-TFBT** molecular films (Figure S1b) show a shift toward the red region at 80 and 120 °C.

As we previously noted,^14,15^ substitution of six fluorine atoms with cyano groups has a remarkable effect on the optoelectronic properties of the resultant materials. The UV–Vis absorption spectra of **EH-TCNBT** and **O-TCNBT** are shown in Figure 1c,d, respectively. Solution spectra were recorded in chloroform, and films were spin-coated from chloroform and annealed at 80 °C. **EH-TCNBT** shows an absorption maximum at 658 nm in solution, which is slightly redshifted at 684 nm in the solid state. The higher energy peak appears at 344 nm in solution, showing a relative increase in intensity without any significant red shift in the solid state. Varying annealing temperatures did not affect the optical spectra (Figure S2a) with the absorption peaks being broad and featureless. The optical band gap was calculated by the onset of absorption spectra at 1.7 and 1.5 eV in solution and solid state, respectively. **O-TCNBT** shows an absorption maximum at 660 nm that is redshifted to 702 nm in the solid state. A clear vibronic peak is apparent at 647 nm that is increased in intensity at higher annealing temperature (Figure S2b). The intensity of the high energy peak at 344 nm relative to the low energy peak is significantly enhanced at higher annealing temperatures. The optical band gap was calculated by the onset of absorption spectra at 1.7 and 1.5 eV in solution and solid state, respectively. Overall, these results suggest that there are significant differences in the solid state ordering of the two materials depending on the nature of the sidechain.

These differences were further explored by examining the thermal behavior of the materials. Gravimetric analysis suggested that all four materials exhibit good thermal stability, with the onset of degradation in nitrogen above 330 °C in all cases (Figure S3). Examination of the melting behavior by DSC showed that the nature of the sidechain had a significant impact. Thus, **O-TFBT** exhibited a sharp melting point at 184 °C, with crystallization on cooling at 124 °C (Figure S4a). Replacement of the fluorine with nitriles resulted in an increase in melting temperature, and **O-TCNBT** exhibited two melt exotherms on heating at 197 and 225 °C. Upon cooling, a glass was formed, which underwent cold crystallization on subsequent heating (Figure S4a). Changing to the branched sidechain changed the thermal behavior drastically. Thus, **EH-TCNBT** only exhibited a glass transition upon heating, around 98 °C, with no sharp melting or crystallization peaks (Figure 4b). Clearly the presence of different stereoisomers and the bulkier 2-ethylhexyl sidechains suppresses crystallization, in agreement with the UV–Vis results.

The electrochemical properties of the fluorinated and cyanated compounds were investigated in dichloromethane solution by cyclic voltammetry (CV) and referenced against ferrocene (Table 1). HOMO and LUMO energy levels for all materials were estimated from the half peaks from the first oxidation (*E*_1/2,ox_) and first reduction potentials (*E*_1/2,red_), respectively. We were unable to perform CV in the solid state due to high solubility of all compounds causing delamination of the films during measurement. The CV spectra of fluorinated precursors are shown in Figure S5. Compound **EH-TFBT** exhibits one quasi-reversible oxidation peak and one quasi-reversible reduction peak with HOMO/LUMO energy levels calculated at −5.5 eV/–3.1 eV, resulting in an electrochemical band gap of 2.4 eV. Substituting with the octyl chain in **O-TFBT** did not have any impact on the electronic properties of the material, with identical oxidation and reduction onsets observed. Cyanation had a significant impact on the electrochemical response compared to their fluorinated analogues, both with respect to their oxidation and reduction. For **EH-TCNBT**, one oxidation peak and two reduction peaks are observed. The first reduction potential is shifted significantly to lower potential compared to the fluorinated starting material, with a second quasi-reversible reduction also appearing (Figure S6). The HOMO/LUMO energy levels were calculated at −6.0 eV/–4.2 eV. Compound **O-TCNBT** showed a similar trend, with only a small alteration on the oxidation potential, which could be related to the error on CV measurements. The HOMO/LUMO energy levels were calculated at −5.9 eV/–4.2 eV (Table 1). The experimental energy levels are in reasonable agreement with theoretical calculations obtained through density functional theory (DFT) using the B3LY/6.31G (d,p) level of theory with the octyl/2-ethylhexyl groups replaced with methyl groups for computational simplicity. HOMO/LUMO energy levels for **TFBT** and **TCNBT** were estimated at −5.2 eV/–3.0 eV and −6.4 eV/–4.4 eV, respectively (Figure S7).

**Table 1:** Energy Levels of EH/O-TFBT and EH/O-TCNBT

	*E*_1/2,ox_, V (HOMO, eV)^a^	*E*_1/2,red_, V (LUMO, eV)^a^	*E*_g_, eV (elec)^a^	λ_max_, nm (sol)^b^	λ_max_, nm (film)^c^	*E*_g_, eV (opt)^d^
**EH-TFBT**	0.7	–1.7	2.4	506	554	1.8
	(−5.5)	(−3.1)				
**O-TFBT**	0.7	–1.7	2.4	510	571	1.8
	(−5.5)	(−3.1)				
**EH-TCNBT**	1.2	–0.6	1.8	658	684	1.5
	(−6.0)	(−4.2)				
**O-TCNBT**	1.1	–0.6	1.7	660	702	1.5
	(−5.9)	(−4.2)				

a Determined by CV in CH_2_Cl_2_ and energy values referenced versus ferrocene/ferrocenium at −4.8 eV.

b Determined by UV–Vis spectroscopy in CHCl_3_.

c Determined by UV–Vis spectroscopy of annealed thin films at 80 °C.

d Determined from the onset wavelength of the absorption spectra in the solid state.

**Figure 2:**
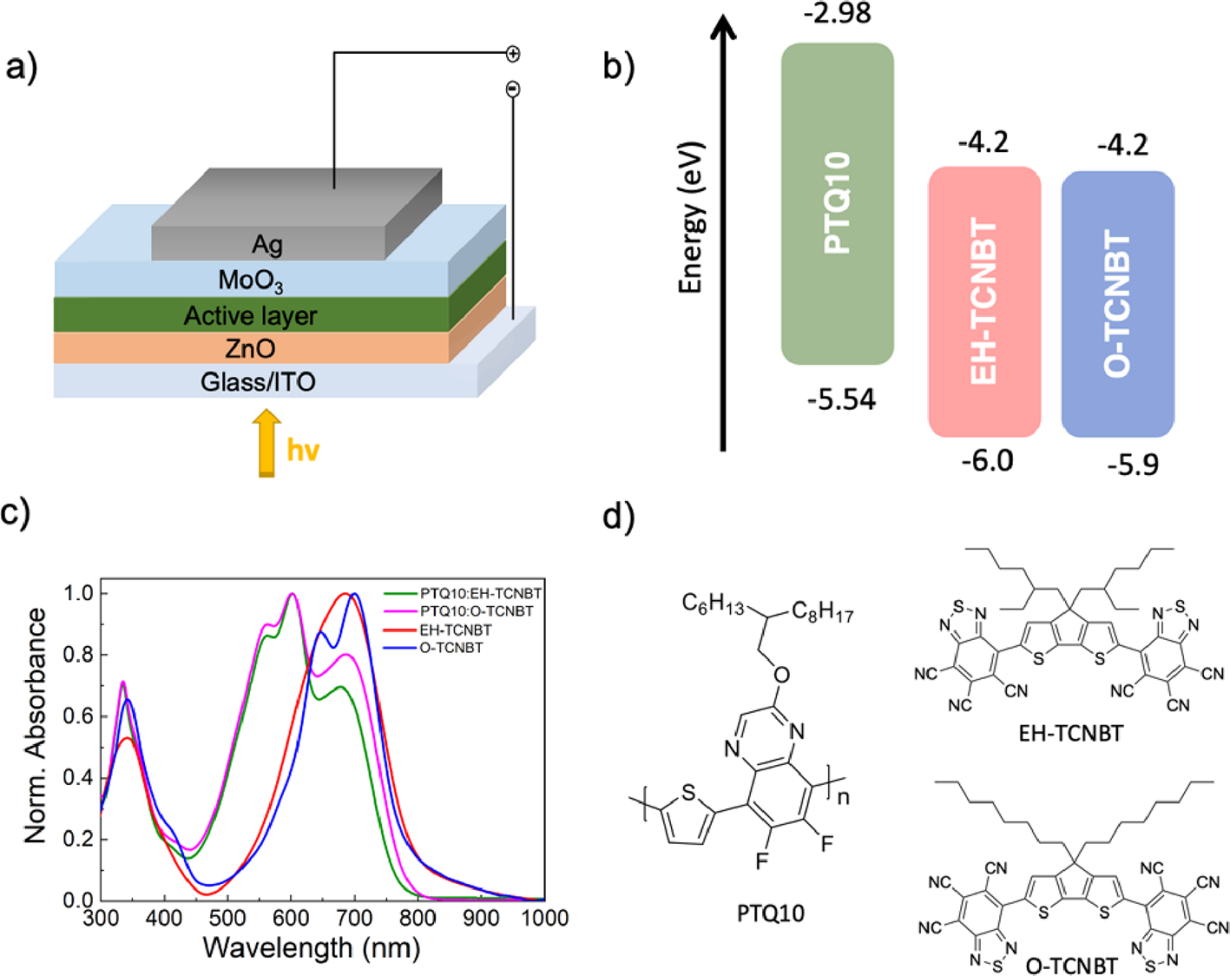
(a) Schematic of the OPD device architecture. (b) Energy levels of PTQ10 were measured by PESA, and **EH/O-TCNBT** were measured by solution CV. (c) UV–Vis absorption of the blends and the acceptors in the solid state, annealed at 100 °C. (d) Structures of polymer donor PTQ10 and small molecule acceptors **EH-TCNBT** and **O-TCNBT**.

**Figure 3:**
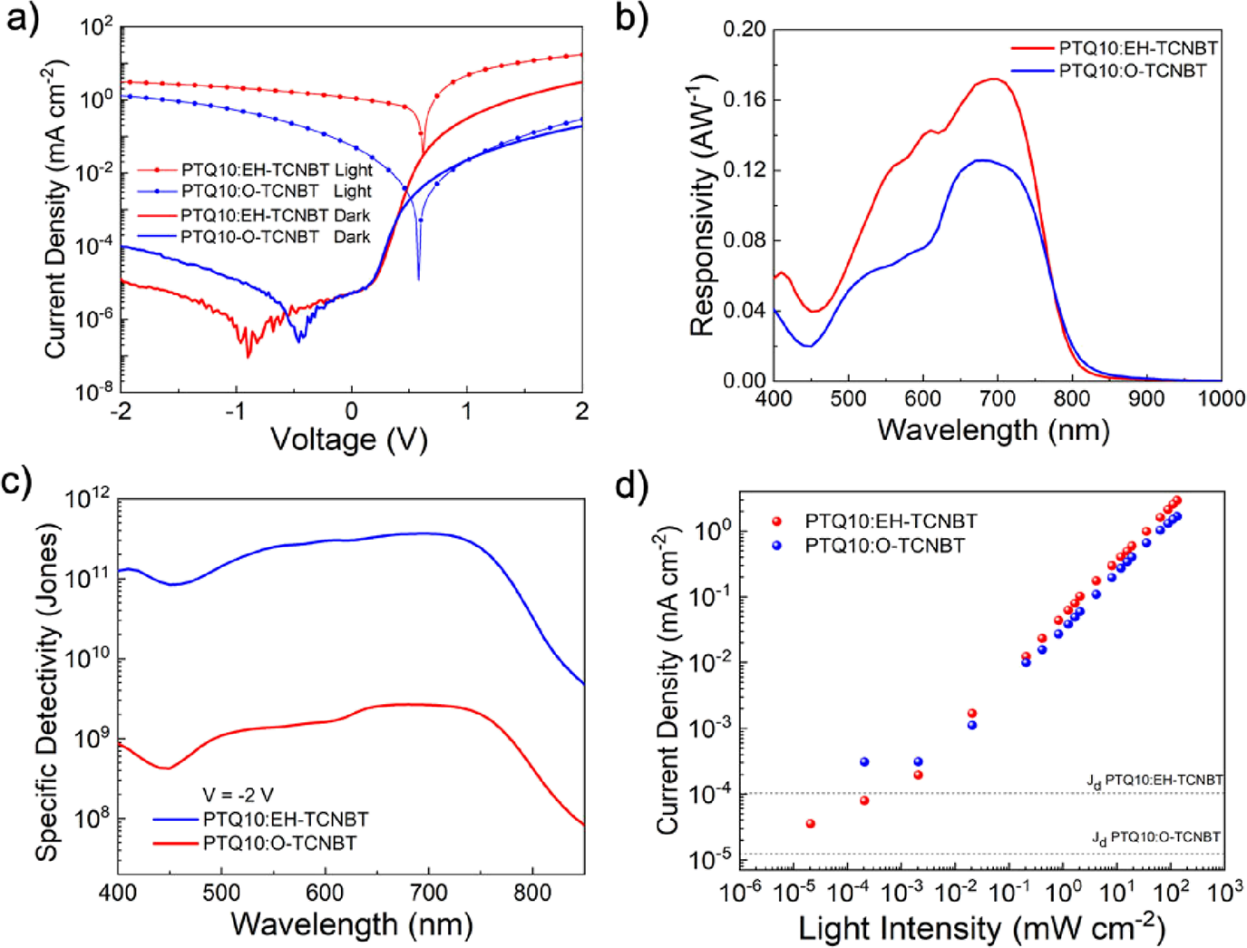
(a) Current density vs voltage response of the optimum devices under 1 sun illumination and in the dark at reversed bias at −2 V, (b) responsivity of the best-performing blends, (c) specific detectivity (D*) at −2 V, and (d) linear dynamic range (LDR).

Organic photodetector performance: **EH-TCNBT** and **O-TCNBT** were investigated as acceptors in blends with the polymer donor PTQ10.^22^ PTQ10 was chosen due to its complimentary absorption spectra as well as it well-matched energy levels (Figure 2). The OPDs were fabricated with an inverted architecture based on indium tin oxide (ITO)/ZnO/active layer/MoOx/Ag at 1:2 donor:acceptor ratio. The UV–Vis absorption spectra of the blends as well as the pure acceptor films are shown in Figure 2c. Here, the vibronic shoulder is clearly apparent in the annealed film of **O-TCNBT** but is suppressed upon blending. The current density–voltage characteristics were measured under no illumination and one sun equivalent illumination (AM 1.5 G) (Figure 3a), and dark current values were extracted at −2 V (Table 2).

**Table 2:** Key Performance Parameters for OPDs Based on PTQ10:**EH-TCNBT** and PTQ10:**O-TCNBT**; Dark Current Density (*J*_d_, Best and Average), Responsivity (*R*), LDR, and Specific Detectivity (D*)^a^

	*J*_d_ (mA cm^–2^)	*R* (A W^–1^) (at λ/nm)	LDR (dB)	D* (Jones)(at λ/nm)
PTQ10:**EH-TCNBT**	1.27 ×10^–5^	0.17	98.4	2.86 × 10^11^
	(1.43 ± 0.15) × 10^–5^	(690)		(750)
PTQ10:**O-TCNBT**	1.01 ×10^–4^	0.13	74.6	2.09 × 10^9^
	(2.93 ± 2.14) × 10^–4^	(680)		(700)

a All Reported at −2 V reverse bias.

PTQ10:**EH-TCNBT**-based OPDs delivered the lowest dark current density of 1.27 ×10^–5^ mA cm^–2^ at −2 V, whereas PTQ10:**O-TCNBT** based OPDs showed *J*_d_ of 1.01 ×10^–4^ mA cm^–2^.

These values compare well with other blends based on A-D-A materials, such as IDTBR, despite the redshifted absorption of the current blend.^12^ It is known that dark currents are usually higher for NIR absorbing materials compared to those that absorb in the visible^23^ due to a variety of factors including large charge recombination.^21−24^ The use of various hole and electron blocking layers in the device has been shown to reduce dark current,^23^ although no such layers are used here. Similar to OPVs, OPDs under illumination should efficiently convert photons to electrons. This can be calculated from the responsivity (*R*), which is related to the external quantum efficiency (EQE) according to , where λ is the wavelength in nm, *h* is the Planck constant, *c* is the speed of light in a vacuum, and *e* is the elementary charge. The responsivity for both acceptor blends is shown in Figure 3b at *V* = −2 V and, in the case of **EH-TCNBT**, is 0.17 AW^–1^ at 690 nm, whereas **O-TCNBT** is lower at 0.13 A W^–1^ at 680 nm. The differences in *R* values are often attributed to the energy offset between the HOMO of the donor and the LUMO of the acceptor.^12^ This energy offset at the interface is necessary for excitons to efficiently split and generate free charge carriers. In our case, both acceptors have similar energy levels, so the difference likely stems from changes in the morphology of the blends.

Specific detectivity (D*) is another important parameter to describe the efficiency of an OPD device and is related to the noise current in the device. D* takes into account both the signal stability and the photodetection ability, identified by the noise current (*i_n_*) and responsivity, respectively, as described by eq 1.
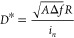
1where *A* is the photodetector active area and Δ*f* is the measurement system bandwidth. The noise current (*i_n_*) is calculated according to eq 2, where *q* is the elementary charge, *i_d_* is the dark current, *k* is the Boltzmann constant, *T* is the temperature, and *R*_shunt_ is the shunt resistance.
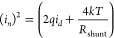
2

The D* of PTQ10:**EH-TCNBT** and PTQ10:**O-TCNBT**-based devices is shown in Figure 3c, and it is similar for both acceptor blends. **EH-TCNBT-**based devices delivered a D* of 2.86 × 10^11^ Jones at 750 nm and, whereas **PTQ10:O-TCNBT** depicted D* = 2.09 × 10^9^ Jones at 700 nm. In addition to detectivity of low light levels, a linear responsivity to different light intensities is preferable especially for image sensor applications, where the difference between minimum and maximum signals is important.^12^ This is expressed by the linear dynamic range (LDR). LDR is defined as the ratio between the photocurrent (*J_ph_ = J_l_* – *J_d_*, where *J_d_* and *J_l_* are the current densities under dark and light conditions, respectively) at high (*j_max_*) and low (*j_min_*) light intensities, according to LDR = 20 log (*j_max_*/*j_min_*). The LDR for PTQ10:**EH-TCNBT** was calculated at 98.4 dB, whereas that for the PTQ10:**O-TCNBT** blend was lower at 74.6 dB, at applied voltage *V* = −2 V (Figure 3d). Notably, the LDR of ∼100 dB obtained for PTQ10:**EH-TCNBT** is outperforming the values obtained for PTQ10-NFA blend devices previously reported.^12^

To have a better understanding of the low *J*_d_ obtained in PTQ10:**EH-TCNBT**, we performed space charge limited current (SCLC) mobility measurements (Figure S8 and Table S1). Compound **EH-TCNBT** exhibits an electron mobility of 1.19 × 10^–6^ cm^2^ V^–1^ s^–1^, and compound **O-TCNBT** has a lower electron mobility of 5.87 × 10^–7^ cm^2^ V^–1^ s^–1^. Charge carrier mobility is often related to trap-assisted recombination. Thus, we calculated the trap state density (*n_t_*) according to *V*_TFL_*= en_t_L*^2^/2ε_0_, where *V*_TFL_, *e*, *L*, and ε_0_ represent the trap-filled limited voltage, the elementary charge, active layer thickness, the relative dielectric constant, and the vacuum permittivity. The lower mobility in O-TCNBT likely relates to the higher trap densities (2.7 × 10^15^ cm^–3^), whereas EH-TCNBT shows lower trap densities of 1.1 × 10^15^ cm^–3^. For the PTQ10:**EH-TCNBT** blend, the electron mobility is very similar to the pristine devices at 1.86 × 10^–6^ cm^2^ V^–1^ s^–1^, whereas in the case of the PTQ10:**O-TCNBT** blend, the electron mobility was reduced to 7.38 × 10^–8^ cm^2^ V^–1^ s^–1^. The hole mobilities of the blends were calculated at 8.28 × 10^–6^ cm^2^ V^–1^ s^–1^ for the PTQ10:**EH-TCNBT** blend and at 7.14 × 10^–8^ cm^2^ V^–1^ s^–1^ for the PTQ10:**O-TCNBT** blend, demonstrating that both blends have balanced mobilities that can reduce charge recombination.^25^ It has been reported that low charge recombination leads to low dark current, which could explain the low values in the dark current in the case of our blends.^26^

To explore further the different *J*_d_ values obtained, we looked into the intermixing of the donor:acceptor components. We calculated the Flory–Huggins interaction parameter, χ, of the binary combinations from contact angle measurements using the relation , where γ1 and γ2 are the surface energy values of individual components in binary blends (Figure S9, Table S2).^27,28^ The χ values for PTQ10:**EH-TCNBT** and PTQ10:**O-TCNBT** binary blends were 6.5 and 9.0, respectively, suggesting that **EH-TCNBT** tends to mix better with PTQ10 compared to the blend PTQ10:**O-TCNBT** (where a higher χ means less interaction between the two components). The poor miscibility in PTQ10:**O-TCNBT** results in low charge carrier mobility in the blend and therefore higher dark current. Finally, we analyzed the short-circuit current density and open-circuit voltage as a function of light intensity to probe charge recombination processes.^29^ We observed a linear relationship of *J*_sc_ vs light intensity suggesting low bimolecular recombination for both blends. Differently, *V*_oc_ vs light intensity plots (Figure S10, Table S2) reveal that PTQ10:**O-TCNBT** suffers from trap-assisted recombination (slope of 1.84*kT*/*q*), whereas trap-assisted recombination is reduced in PTQ10:**EH-TCNBT** blends (slope of 1.52*kT*/*q*). In line with previous reports, we ascribed the low dark currents in PTQ10:**EH-TCNBT** to reduced trap-assisted recombination.^26^

## Conclusions

Here, we present the synthesis and optoelectronic characterization of two novel electron-accepting materials incorporating strongly electron-withdrawing end groups, **EH-TCNBT** and **O-TCNBT**. Both materials were synthesized from their fluorinated precursors (**EH-TFBT** and **O-TFBT)** that underwent sixfold nucleophilic aromatic substitution with cyanide. The two materials differ in the nature of the solubilizing alkyl chain, branched 2-ethyl hexyl (EH), compared to a linear octyl (O) chain. The nature of the sidechain was found to strongly affect the ordering of the resulting materials, with the linear compound exhibiting well-defined melting behavior and clear signs of thin-film aggregation by UV–Vis spectroscopy. The branched material in contrast appeared largely amorphous due to a combination of the presence of the bulky sidechains and the presence of different diastereomers. The HOMO/LUMO energy levels of the fluorinated materials were estimated at −5.5 eV/–3.1 eV and for cyanated at −6.0 eV/–4.2 eV. Both **EH-TCNBT** and **O-TCNBT** were blended with the low-cost polymer donor PTQ10 to form a BHJ active layer for OPD application. Both materials performed well in organic photodetectors with the PTQ10:**EH-TCNBT** exhibiting lower dark current and higher responsivity compared to PTQ10:**O-TCNBT**, with an excellent linear dynamic range. The lower performance of the PTQ10:**O-TCNBT** blend results mainly from its lower charge mobility. We believe that these results demonstrate the usefulness of highly cyanated benzothiadiazoles for near-infrared detection.

## Experimental Section

### Methods

The detailed information for materials and equipment can be found in the Supporting Information.

### Synthesis

The synthesis of compounds **1**–**3** is described in the Supporting Information.

Synthesis of 7,7′-(4,4-bis(2-ethylhexyl)-4*H*-cyclopenta[2,1-*b*:3,4-*b*′]dithiophene-2,6-diyl) bis(4,5,6-trifluorobenzo[*c*][1,2,5]thiadiazole) (**EH-TFBT**):

4,5,6-Trifluoro-2,1,3-benzothiadiazole^14^ (452 mg, 2.37 mmol), Pd_2_(dba)_3_·CHCl_3_ (50 mg, 5%), tris(*o*-anisyl) phosphine (31 mg, 0.1 mmol), pivalic acid (30 mg, 0.3 mmol)), and cesium carbonate (933 mg, 2.9 mmol) were added in a sealed vial and purged with nitrogen. A degassed solution of **3a** (535 mg, 0.95 mmol) in anhydrous toluene (3 mL) was added, and the mixture was heated to 120 °C for 12 h. After cooling, toluene was removed under reduced pressure and the residue was dissolved in CH_2_Cl_2_. The organic phase was washed with water and brine, and the crude product was purified using column chromatography [eluent: hexane/CH_2_Cl_2_ 3:1 (v:v)] and triturated with ice-cold methanol (15 mL). The product was isolated as a deep red solid (236 mg, 0.3 mmol, 32%); m.p. (DSC) 121.4 °C; ^1^H NMR (400 MHz, CDCl_3_): δ = 8.16 (t, *J* = 7.0 Hz, 2H), 2.06 (t, *J* = 4 Hz, 4H), 1.02–0.96 (m, 18H), 0.66–0.60 (m, 12 H) ppm; ^19^F-{H} NMR (376 MHz, CDCl_3_): δ = −127.3 (d, *J* = 18 Hz), −146.3 (d, *J* = 18 Hz), −153.2 (t, *J* = 18 Hz) ppm; UV/Vis (CHCl_3_): λ_max_ (ε): 506 nm (38,392 M^–1^ cm^–1^); MS (MALDI-TOF): isotopic cluster at *m*/*z* 779.18 [M^+^].

Synthesis of 7,7′-(4,4-dioctyl-4*H*-cyclopenta[2,1-*b*:3,4-*b*′]dithiophene-2,6-diyl) bis(4,5,6-trifluorobenzo[*c*][1,2,5]thiadiazole) (**O-TFBT**):

An identical procedure to the synthesis of **EH-TFBT** was used, starting with **3b** (1.25 g, 2.2 mmol). The product was isolated as a red solid (0.4 g, 0.5 mmol, 23%); m.p. (DSC) 124.7 °C; ^1^H NMR (400 MHz, CDCl_3_): δ = 8.15 (s, 2H), 2.00 (t, *J* = 7 Hz, 4 H) 1.21–1.05 (m, 24H), 0.79 (t, *J* = 4 Hz, 12 H) ppm; ^19^F-{H} NMR (376 MHz, CDCl_3_): *δ* = −126.8 (d, *J* = 18 Hz), −146.2 (d, *J* = 18 Hz), −153.2 (t, *J* = 18 Hz) ppm; UV/Vis (CHCl_3_): λ_max_ (ε): 508 nm (44,278 M^–1^ cm^–1^); MS (MALDI-TOF): isotopic cluster at *m*/*z* 779.28 [M^+^].

Synthesis of 7,7′-(4,4-bis(2-ethylhexyl)-4*H*-cyclopenta[2,1-*b*:3,4-*b*′]dithiophene-2,6-diyl) bis(benzo[*c*][1,2,5]thiadiazole-4,5–6-tricarbonitrile) (**EH-TCNBT**):

Compound **EH-TFBT** (320 mg, 0.41 mmol), KCN (**Caution**: highly toxic, handle with care; 190 mg, 2.90 mmol), and 18- crown-6 (10 mg, 0.04 mmol) were added in a microwave vial and purged with nitrogen. Anhydrous DMF (10 mL) was added, and the mixture was heated at 50 °C for 12 h. The reaction mixture was cooled to RT, added to water (100 mL), and extracted with DCM (100 mL). The aqueous extracts were treated with ammonia solution (28%) to destroy any residual cyanide present. The organic phase was washed with water (100 mL) and brine (100 mL) and finally dried over Na_2_SO_4_. The solvent was removed under reduced pressure, and the crude product was purified by column chromatography over silica [eluent: CH_2_Cl_2_–hexane 5:1 (v:v)]. The product was further purified by recycling GPC (eluent: chloroform) and a blue solid was isolated (212 mg, 0.26 mmol, 63%); ^1^H NMR (400 MHz, CDCl_3_): δ 8.61 (t, *J* = 4.0 Hz, 2H), 2.20–2.09 (m, 4H), 1.04–0.96 (m, 15H), 0.68–0.62 (m, 14H) ppm; ^13^C NMR (101 MHz, CDCl_3_): *δ* 162.7, 153.1, 152.2, 147.9, 137.6, 137.0, 129.7, 122.5, 115.7, 113.0, 111.9, 108.5, 106.5, 55.4, 43.4, 35.7, 34.3, 28.6, 27.4, 22.9, 14.1, 10.7 ppm; UV/Vis (CHCl_3_): λ_max_ (ε): 658 nm (50,770 M^–1^ cm^–1^); MS (MALDI-TOF): isotopic cluster at *m/z* 820.8 [M^+^].

Synthesis of 7,7′-(4,4-dioctyl-4*H*-cyclopenta[2,1-*b*:3,4-*b*′]dithiophene-2,6-diyl) bis(benzo[*c*][1,2,5]thiadiazole-4,5–6-tricarbonitrile) (**O-TCNBT**):

An identical procedure to the synthesis of **EH-TCNBT** was used, starting with **O-TFBT** (300 mg, 0.39 mmol) The product was isolated as a blue solid (196 mg, 0.24 mmol, 61%). m.p. (DSC) 225 °C; ^1^H NMR (400 MHz, CDCl_3_): δ 8.57 (s, 2H), 2.08–2.03 (m, 4H), 1.17–1.09 (m, 24 H), 0.81 (t, *J* = 8 Hz, 6H) ppm; ^13^C NMR (101 MHz, CDCl_3_): δ 163.1, 153.0, 152.1, 147.7, 137.8, 137.4, 129.2, 122.3, 115.8, 113.0, 111.9, 108.3, 106.6, 55.2, 37.5, 31.9, 30.0, 29.4, 29.3, 25.2, 22.7, 14.2 ppm; UV/Vis (CHCl_3_): λ_max_ (ε): 660 nm (83,436 M^–1^ cm^–1^); MS (MALDI-TOF): isotopic cluster at *m/z* 821.2 [M^+^].

### OPD Fabrication

PTQ10:**EH-TCNBT** and PTQ10:**O-TCNBT** organic photodetectors were fabricated using an inverted structure (glass/ITO/ZnO/active layer/MoO_3_ (10 nm)/Ag (100 nm)). The indium tin oxide (ITO, 15 Ω sq.^–1^) was pre-patterned on the glass substrates (12 mm × 12 mm). For inverted structure devices, a 40 nm-thick ZnO layer was deposited on the ITO by 4000 rpm, 40 s spin-coating using a zinc acetate dihydrate precursor solution (219 mg of zinc acetate dihydrate precursor dissolved in 60.4 μL of 1-ethanolamine and 2 mL of 2-methoxyethanol followed by annealing at 180 °C for 10 min. The donor and acceptor were blended in a 1:1 ratio (wt/wt) in a 20 mg/mL concentration in chloroform. The solutions were stirred overnight in a nitrogen glovebox at room temperature and heated at 40 °C for 20 min before spin-coating. The active layer solution was spin coated on the ZnO from a warm solution, at different spin speeds ranging from 1000 to 2000 rpm for 40 s. The active layer was annealed at 100 °C for 5 min. For the thermal evaporation, a 10 nm MoO_3_ and a 100 nm Ag layer were sequentially deposited.

## Supporting Information Available

**EH-TCNBT** and **O-TCNBT** synthesis and characterization, device fabrication and characterization, ^1^H, ^19^F, and ^13^C NMR spectra; electrochemical cyclic voltammograms; UV–Vis spectra as a function of temperature; TGA and DSC; DFT calculations; SCLC of hole and electron-only devices; contact angle measurements; light intensity measurements; calculation of Flory–Huggins interaction parameter; AFM (PDF)
