# Supplementary material for: Low Dark Current Organic Photodetectors Utilizing Highly Cyanated Non-fullerene Acceptors
Source: ACS Appl Mater Interfaces. 2022 Aug 16;14(34):39141–8. doi: 10.1021/acsami.2c10197 (PMC9437869; doi:10.1021/acsami.2c10197)
Supplement: Supplementary file 1 — am2c10197_si_001.pdf [file am2c10197_si_001.pdf]

Supporting Information

Low dark current organic photodetectors utilizing highly cyanated non-fullerene acceptors

Panagiota Kafourou[1], Zhuoran Qiao[1], Máté Tóth[1], Filip Aniés[1], Flurin Eisner[2], Nicola Gasparini[1], Martin Heeney[1]

n.gasparini@imperial.ac.uk; m.heeney@imperial.ac.uk

[1]Department of Chemistry and Centre for Processable Electronics, Imperial College London, London, W12 0BZ, UK.

[2]Department of Physics, and Centre for Processable Electronics, Imperial College London, London, SW7 2AZ, UK.

Table of Contents

Methods ***2***

Atomic force microscopy (AFM) ***2***

Synthesis ***3***

Optical Properties ***5***

Thermal Properties ***6***

Electronic Properties & Theoretical Calculations ***7***

OPD performance ***8***

NMR Spectra ***12***

References ***19***

Methods

Methods

Purification of products EH/O-TFBT and EH/O-TCNBT was conducted with recycling GPC from Japan Analytical Industry Co, Ltd; the eluent used was chloroform. The system consisted of a high pressure liquid chromatography apparatus (JAI LaboACE LC 5060 series) equipped with a pump (P-LA60, flow rate 10 ml min−1), a UV detector (UV-VIS4ch LA, λ = 210 nm, 254 nm, 330 nm, 400 nm) and two columns (Jaigel 2HR and 2.5HR, inner diameter 20 mm × length 600 mm each). Nuclear magnetic resonance (NMR) spectra were recorded on Bruker AV-400 (400 MHz) spectrometers in CDCl3 using the residual solvent resonance of o-DCB-d4. Spectra were recorded at r.t unless stated otherwise. UV-Vis spectra in chloroform solution were recorded in a UV-1601 Shimadzu UV-vis spectrometer. Films were prepared by spin-coating from 20 mg mL^{-1} solutions in chloroform onto glass substrates.

Atomic force microscopy (AFM)

The surface morphology of the films was studied using tapping-mode AFM on an Agilent 5500AFM system. The resonant frequency of the cantilever was approximately 270kHz with a force constant of 40 N m^{-1}. The images were analysed using Gwyddion software, with the same processing procedures applied to all images.

Electrochemical characterisation

Cyclic and square-wave voltammograms were recorded using a Metrohm Autolab PGSTAT101 potentiostat/galvanostat. The experimental setup consisted of an Ag/Ag+ reference electrode, a platinum wire counter electrode and a platinum working electrode, and all measurements were carried out under nitrogen at room temperature. Measurements were performed in anhydrous, degassed solutions of CH_2Cl_2 with tetrabutylammonium hexafluorophosphate (0.1 M) electrolyte. Potentials were referenced to those of ferrocene when a ferrocene/ferrocenium reference redox system of 4.8 eV below the vacuum level was used as an internal standard, and the conversion from electrochemical potentials to electron volts was done using the formula $E(eV) = -Er_{edox} - 4.8$ eV.[1] Any solvent effects were neglected.

Density functional theory (DFT) calculations

DFT calculations were conducted using Gaussian 09 software on the Imperial College High-Performance Computing Service.[2] All simulations were carried out on single molecules in the

gas phase at the B3LYP level of theory with the basis set 6-31G(d,p).[3] Alkyl side chains were replaced by methyl groups to reduce the computation time. Structures were optimized to a local minimum energy conformation.

Synthesis

Synthesis of **2a**:[4] Compound **1** (1.0 g, 5.6 mmol) and potassium tert-butoxide (2.0 g, 18 mmol) were purged with nitrogen followed by the addition of degassed, anhydrous DMSO (30 mL). The reaction mixture was stirred at 60 °C for 1 hr. 2-Ethylhexyl bromide (3.32 g, 17 mmol) was then added dropwise and the reaction heated at 80 °C for 12 h. The mixture was left to cool to room temperature and the mixture was decanted in 150 mL of water, followed by extraction with hexane (2 x 200 mL) and was further washed with water (100 mL) and brine (100 mL). The organic phase was dried ($MgSO_4$) and the solvent removed under reduced pressure to afford the crude product. Purification using column chromatography (eluent: petroleum ether) yielded a yellow, viscous oil (1.67 g, 4.10 mmol, 74 %). ^1H NMR ($CDCl_3$, 400 MHz): δ 7.11 (d, J = 4 Hz, 2H), 6.93 (dt, J = 4, 2 Hz, 2H), 1.92-1.85 (m, 4H), 1.83-0.86 (m, 18 H), 0.79-0.74 (m, 6H), 0.59 (overlapping t, J = 8 Hz, 6H) ppm; ^{13}C NMR ($CDCl_3$, 101 MHz): δ 157.7, 136.9, 124.1 (d), 122.5, 122.4 (d), 53.4, 43.4, 35.1, 34.3, 28.7, 27.4, 22.9, 14.2, 10.8 ppm.

Synthesis of **3a**.[5] To a solution of **2a** (1.60 g, 4.0 mmol) in THF (36 mL) and DMF (18 mL) was added N-bromosuccinimide (1.55g, 8.7 mmol) under nitrogen. The reaction was stirred at room temperature for 30 min in the absence of light. After reaction completion, which was monitored by TLC (eluent: petroleum ether), sat. Na_2SO_3 was added followed by addition of water (50 mL). The product was extracted with hexane (2 x 50 mL) and dried over $MgSO_4$. Removal of the solvent under reduced pressure afforded the product as a yellow oil (1.83 g, 3.3 mmol, 83 %). ^1H NMR ($CDCl_3$, 400 MHz): δ 6.93 (t, J = 4 Hz, 2H), 1.85 – 1.75 (m, 4H), 1.04 – 0.85 (m, 18H), 0.80-0.77 (m, 6 H), 0.62 (overlapping, J = 8 Hz, 6H) ppm; ^{13}C NMR ($CDCl_3$, 101 MHz): δ 155.7, 136.8, 125.4 (t), 110.9 (t), 55.1, 43.2, 35.3, 34.2, 28.7, 27.5, 23.0, 14.3, 10.8 ppm.

Synthesis of **2b**. Compound **1** (3.0 g, 17.0 mmol) and potassium *tert*-butoxide (6.0 g, 10.0 mmol) were purged with nitrogen followed by the addition of degassed, anhydrous DMSO (50

mL). The reaction mixture was stirred at 80 °C for 1 hr. 1-Bromooctane (9.66 g, 50.0 mmol) was then added dropwise and the reaction was heated at 80 °C for 12 h. The mixture was left to cool to room temperature and the mixture was decanted in 150 mL of water, followed by extraction with hexane (2 x 200 mL) and was further washed with water (100 mL) and brine (100 mL). The organic phase was dried ($MgSO_4$) and the solvent removed under reduced pressure to afford the crude product. Purification using column chromatography (eluent: petroleum ether) yielded a yellow, viscous oil (5.15 g, 13.0 mmol, 76 %). ^{1}H NMR ($CDCl_3$, 400 MHz): δ 7.14 (d, J = 4 Hz, 2H), 6.93 (d, J = 4 Hz, 2H), 1.83-1.79 (m, 4H), 1.16-1.12 (m, 24 H), 0.84 (t, J = 8 Hz, 6H) ppm; ^{13}C NMR ($CDCl_3$, 101 MHz): δ 158.3, 136.6, 124.6, 121.8, 53.4, 37.9, 32.0 (d), 30.2, 29.5, 29.4, 22.8, 14.3 ppm.

Synthesis of **3b.** To a solution of **2b** (2.0 g, 5.0 mmol) in THF (45 mL) and DMF (25 mL) was added *N*-bromosuccinimide (2.0 g, 11.0 mmol) under nitrogen. The reaction was stirred at r.t. for 30 min in the absence of light. After reaction completion, which was monitored by TLC (eluent: petroleum ether), sat. Na_2SO_3 was added followed by addition of water (50 mL). The product was extracted with hexane (2 x 50 mL) and dried over $MgSO_4$. Removal of the solvent under reduced pressure afforded the product as a yellow oil (2.34 g, 4.20 mmol, 84%). ^{1}H NMR ($CDCl_3$, 400 MHz): δ 6.92 (s, 2H), 1.77 – 1.73 (m, 4H), 1.27 – 1.14 (m, 24H), 0.86 (t, J = 5 Hz, 6H) ppm.

Optical Properties

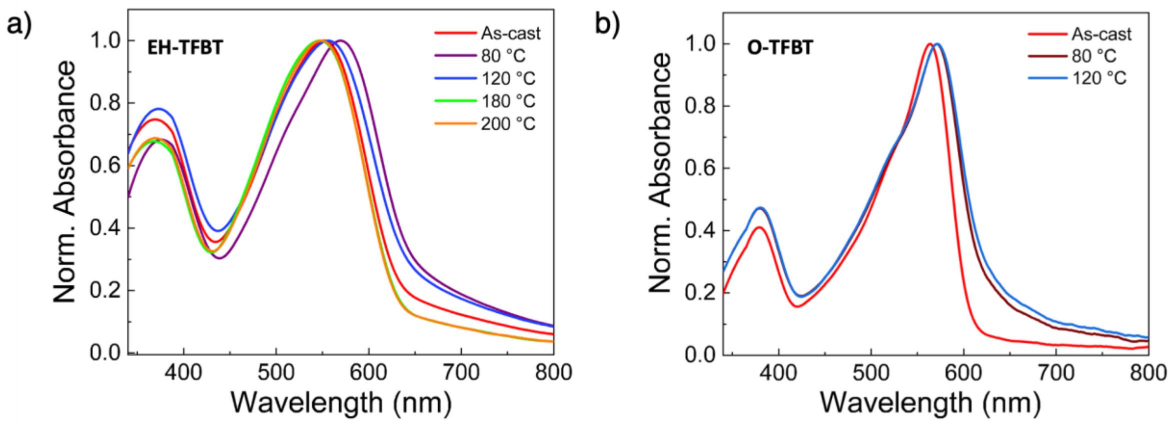

Figure S1: Thin-film UV-Vis absorption spectra of a) EH-TFBT and b) O-TFBT as a function of temperature.

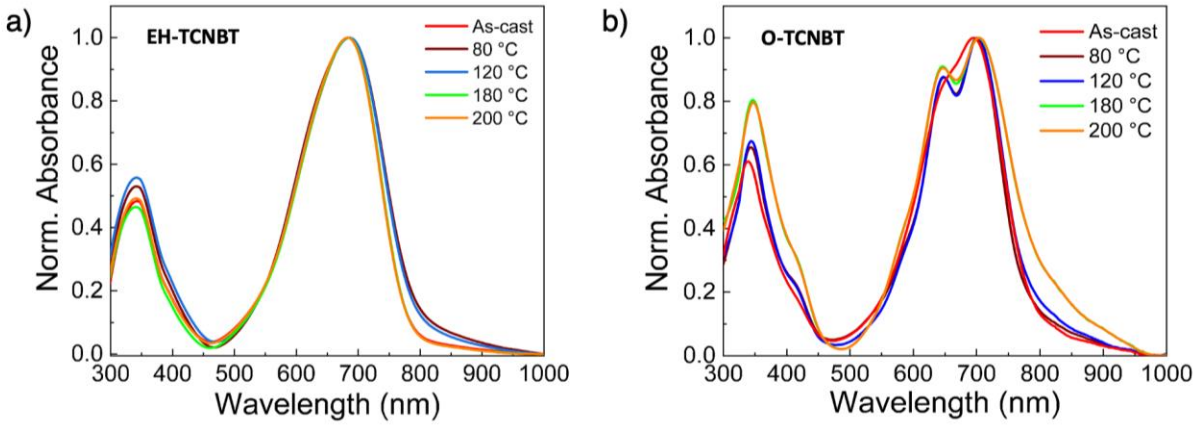

Figure S2: Thin-film UV-Vis absorption spectra of a) EH-TCNBT and b) O-TCNBT as a function of temperature.

Thermal Properties

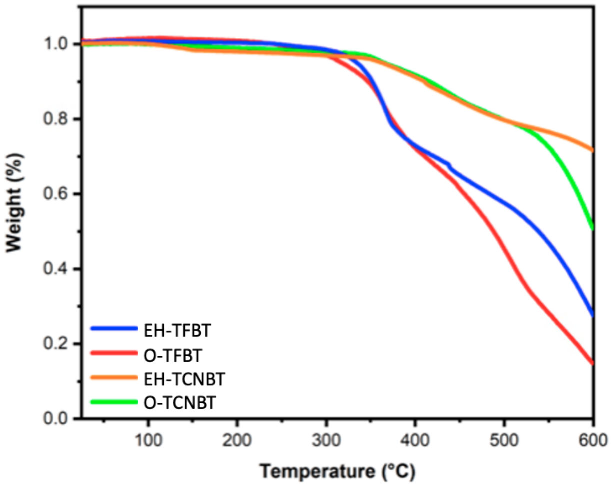

Figure S3: TGA results as a function of temperature between 25 and 600 ºC, at a heating rate of 10 ºC/min and an airflow rate of 50 mL/min.

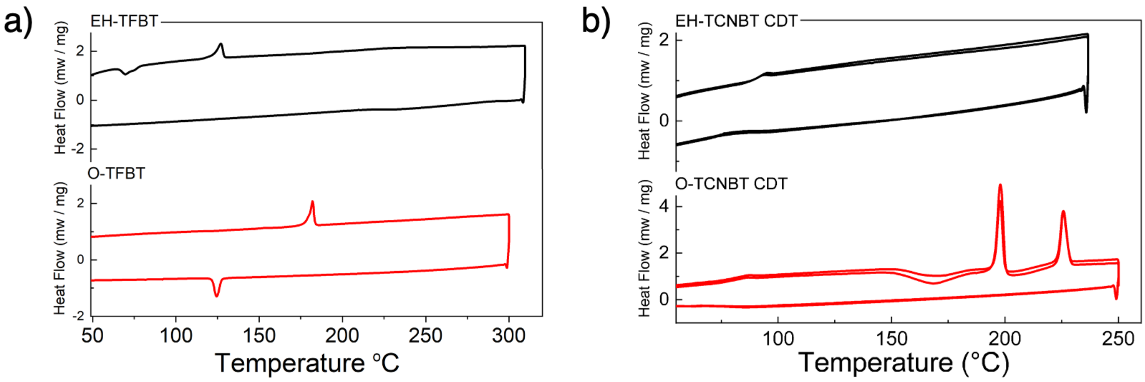

Figure S4: DSC curves for compounds a) EH/O-TFBT, b) EH/O-TCNBT with heating and cooling rates of 10 °C min^{-1}.

Electronic Properties & Theoretical Calculations.

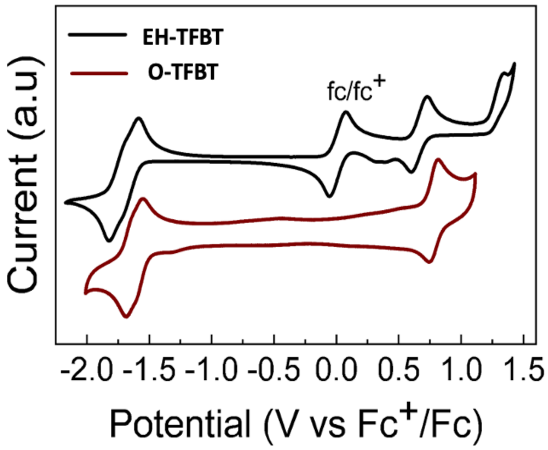

Figure S5: Cyclic voltammograms in DCM-[n-Bu_4N]PF_6 solution (0.1 M) at 100 mVs^{-1} scan rate of **EH-TFBT** (top) and plotted with fc/fc+ into the solution **O-TFBT** (bottom) plotted without fc/fc+ into the solution.

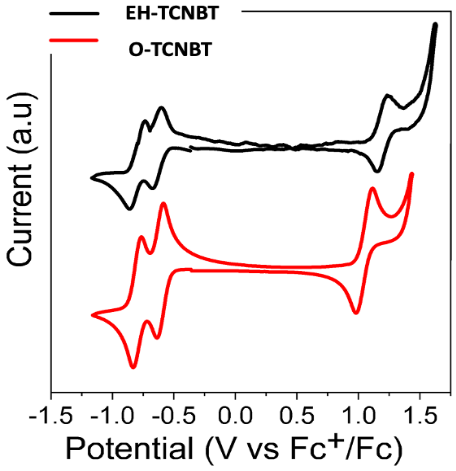

Figure S6: Cyclic voltammograms in DCM-[n-Bu_4N]PF_6 solution (0.1 M) at 100 mVs^{-1} scan rate of **EH-4.5a** (top) and **O-4.5b** (bottom). Potentials were referenced against fc/fc+.

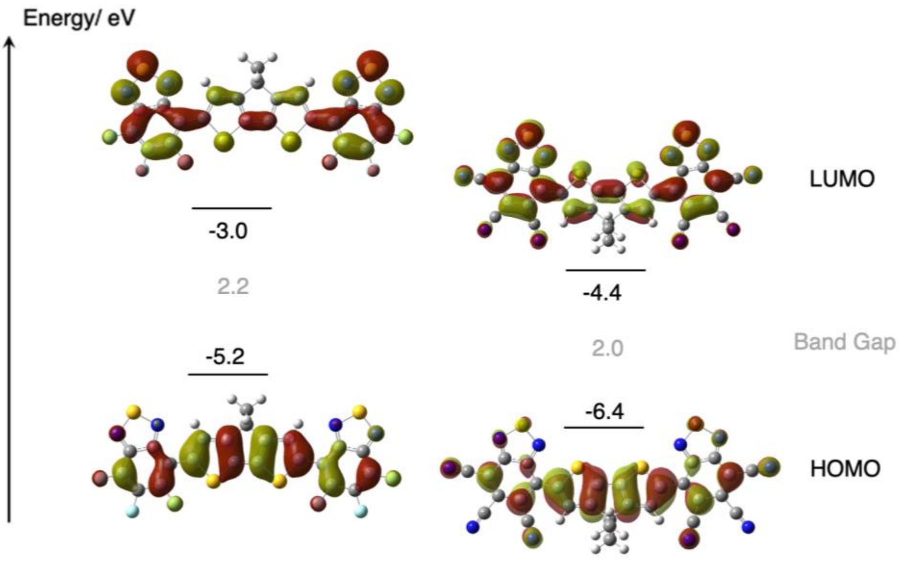

Figure S7: Orbital visualizations of the minimized energy structures (top view) **TFBT**, left, and **TCNBT**, right.

OPD performance

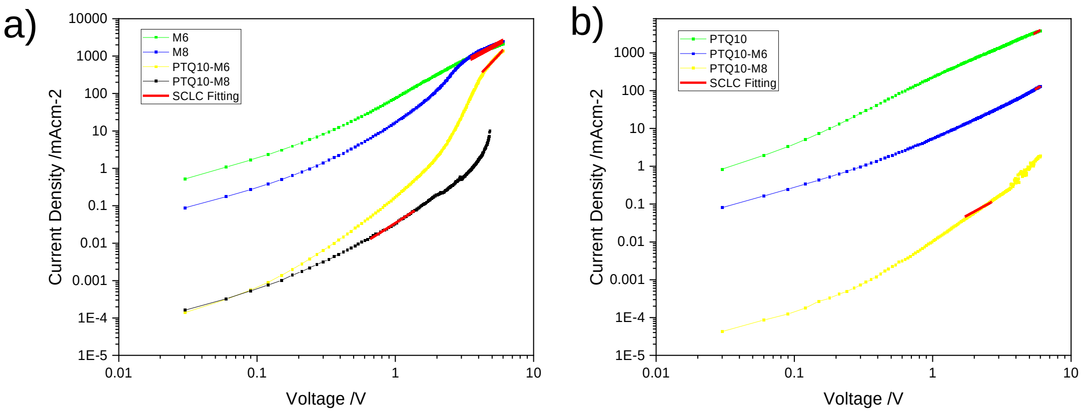

Figure S8: Charge carrier mobility form SCLC* a) electron only and b) hole only, where M6 refers to EH-TCNBT and M8 refers to O-TCNBT.

**Using Mott-Gurney equation:* $J = \frac{9}{8}\varepsilon\varepsilon_0\mu_0\frac{(V-V_{bi})^2}{L^3}exp\left(0.89\gamma\sqrt{\frac{V-V_{bi}}{L}}\right)$ *and Poole-Frenkel expression:* $\mu = \mu_0\, exp(\gamma\sqrt{E})$ *where* $E = 1\times10^5\ Vcm^{-1}$.

Table S1: Charge carrier mobility calculated from SCLC.

	μ_0	γ	Hole mobility**	μ_0	γ	Electron mobility**
**PTQ10**	1.07×10^{-8}	0	1.07×10^{-4}			
**EH-TCNBT**				1.19×10^{-10}	0	1.19×10^{-6}
**O-TCNBT**				5.87×10^{-11}	0	5.87×10^{-7}
**PTQ10:EH-TCNBT**	8.28×10^{-10}	0	8.28×10^{-6}	1.42×10^{-10}	8.56×10^{-4}	1.86×10^{-6}
**PTQ10:O-TCNBT**	7.14×10^{-12}	0	7.14×10^{-8}	7.38×10^{-12}	0	7.38×10^{-8}

** Mobility unit: $cm^2V^{-1}s^{-1}$.

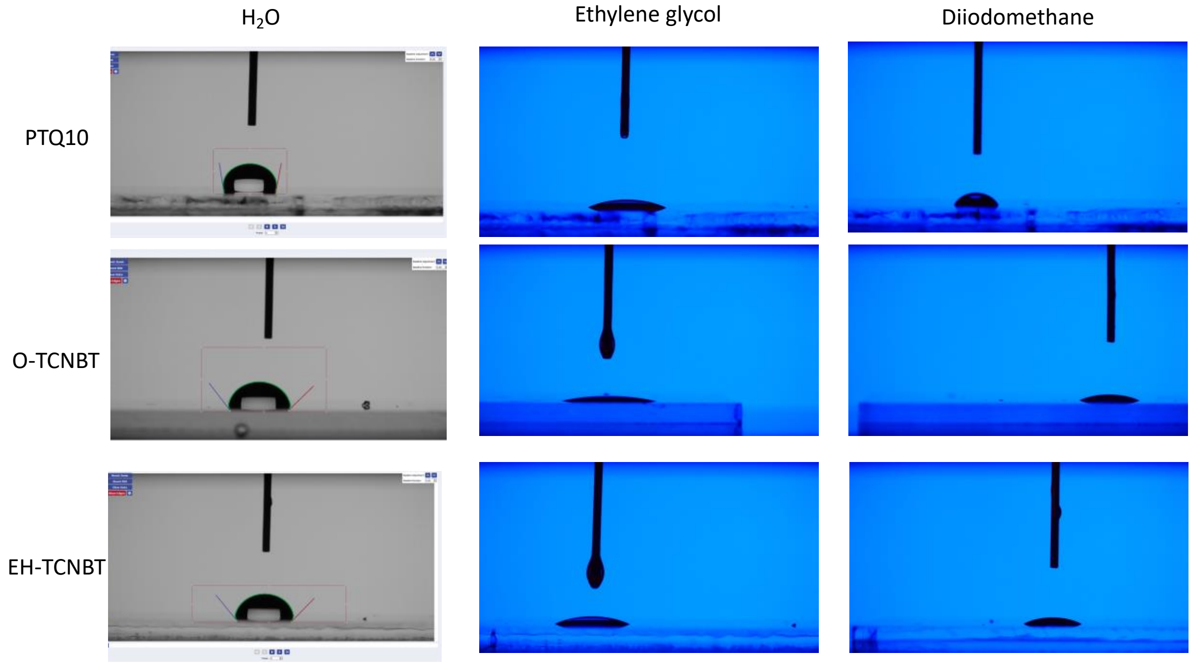

Figure S9: Photographs for contact angle measurement for polymer PTQ10 and acceptors O-TCNBT and EH-TCNBT.

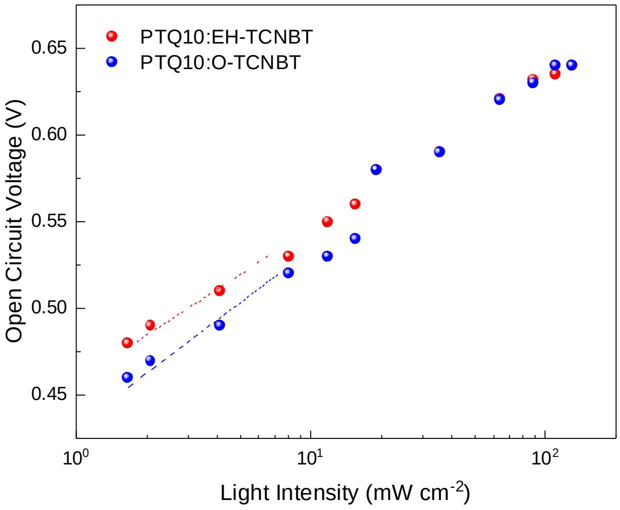

Figure S10: Open-circuit voltage as a function of light intensity to probe charge recombination processes for PTQ10:EH-TCNBT and PTQ10:O-TCNBT blends.

Table S2: Flory−Huggins interaction parameter, χ, of the binary combinations from contact angle measurements using the relation $\chi_{1,2} \propto (\sqrt{\gamma_1} - \sqrt{\gamma_2})^2$ and slope calculated from open-circuit voltage as a function of light.

	**Flory−Huggins interaction parameter (χ)**	**Slope**
PTQ10:EH-TCNBT	6.5	1.51 kT/q
PTQ10:O-TCNBT	9.5	1.76 kT/q

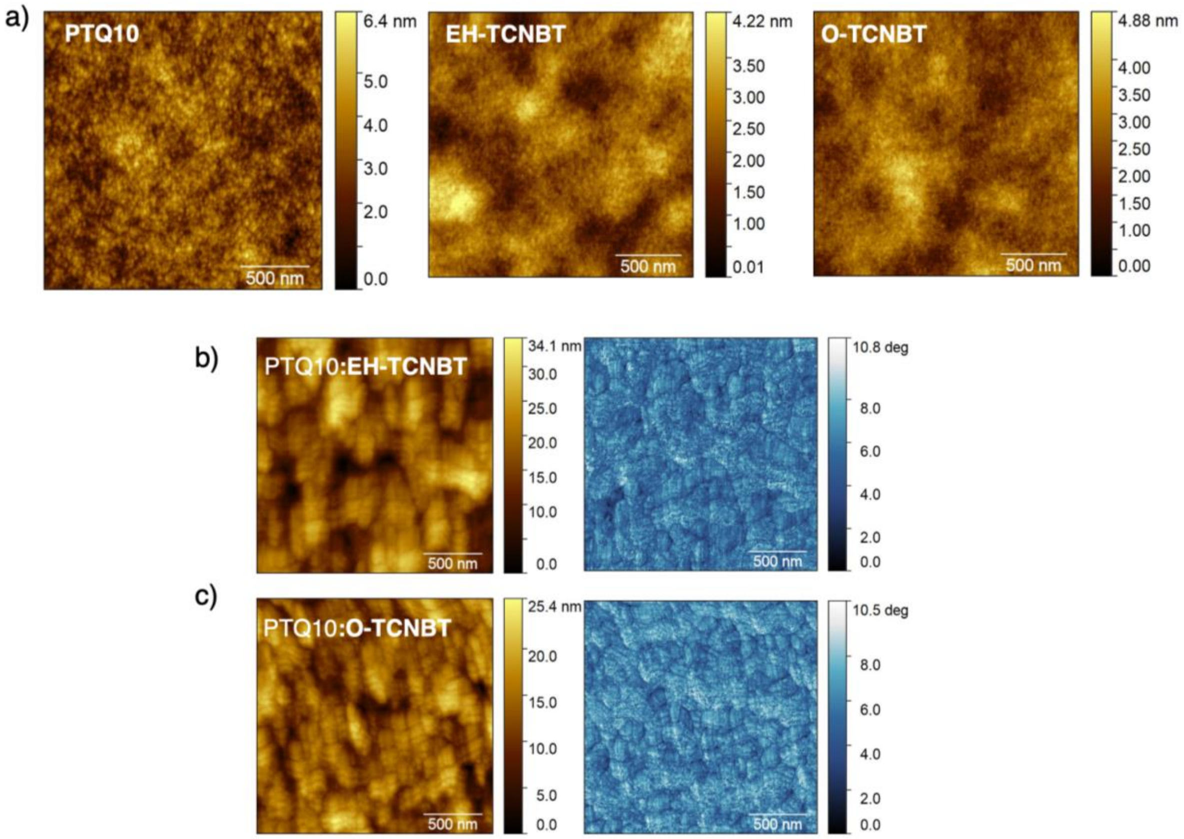

Figure S11: AFM images of a) PTQ10 (RMS = 0.76 nm), EH-TCNBT (RMS = 0.62 nm) and O-TCNBT (RMS = 0.55 nm); b) PTQ10:EH-TCNBT-based blend (RMS = 5.4 nm) and c) PTQ10:O-TCNBT-based blend (RMS = 3.5 nm).

NMR Spectra

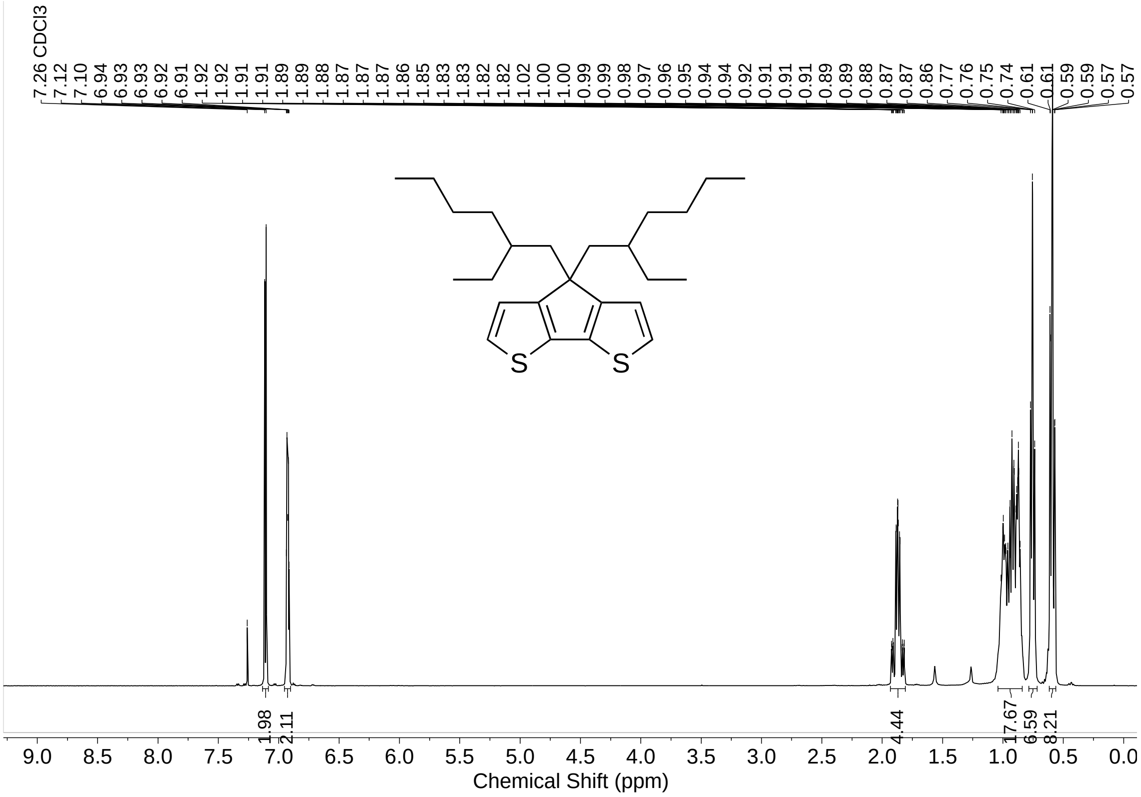

Figure S12: ^{1}H NMR spectrum of compound **2a** in $CDCl_3$.

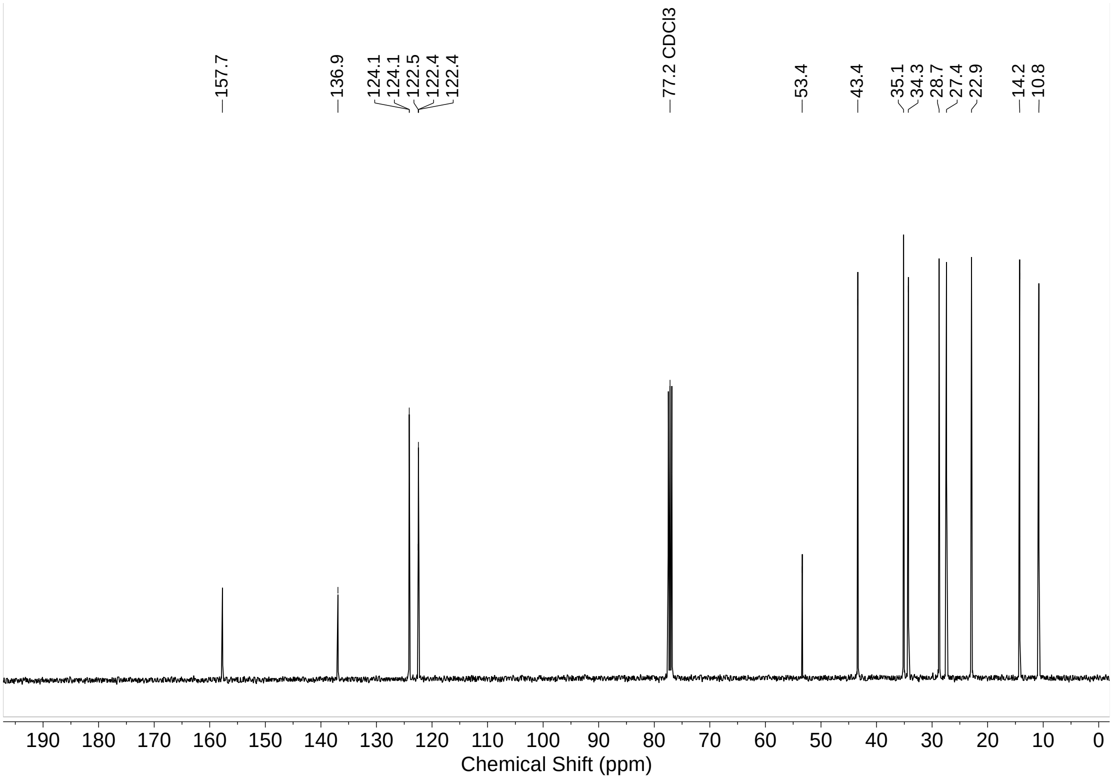

Figure S13: ^{13}C NMR spectrum of compound **2a** in $CDCl_3$.

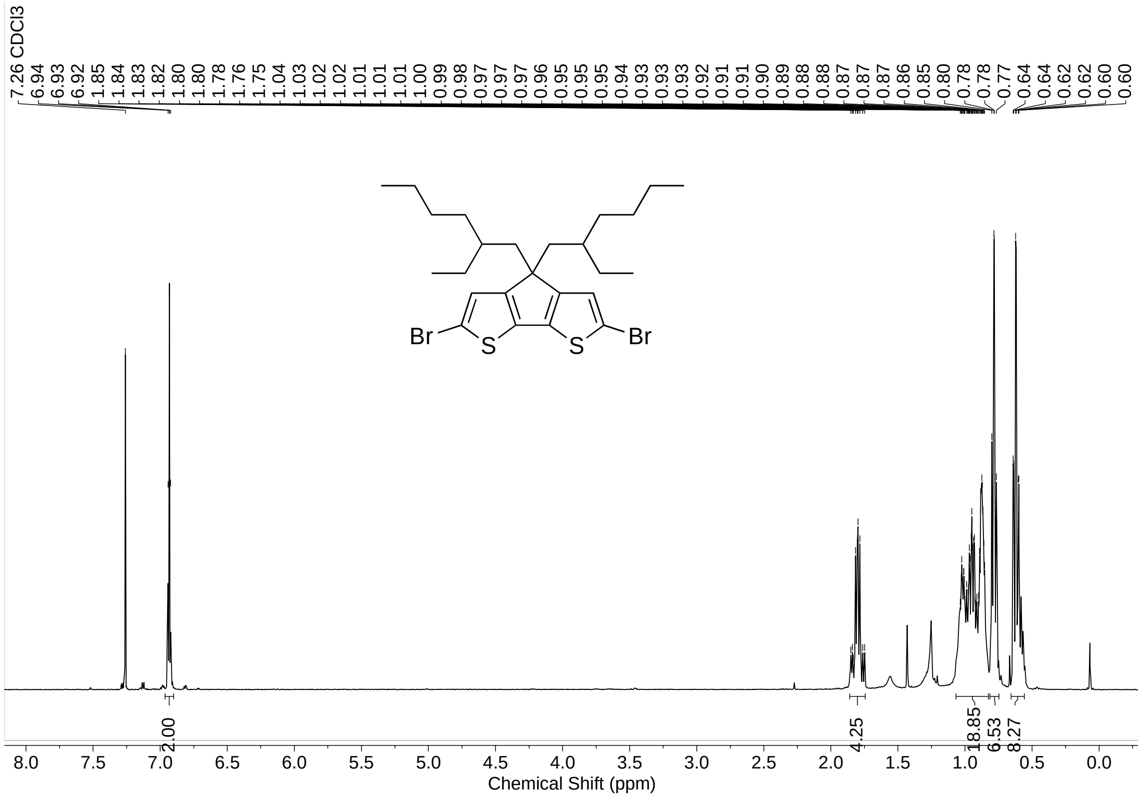

Figure S14: ^{1}H NMR spectrum of compound **3a** in $CDCl_3$.

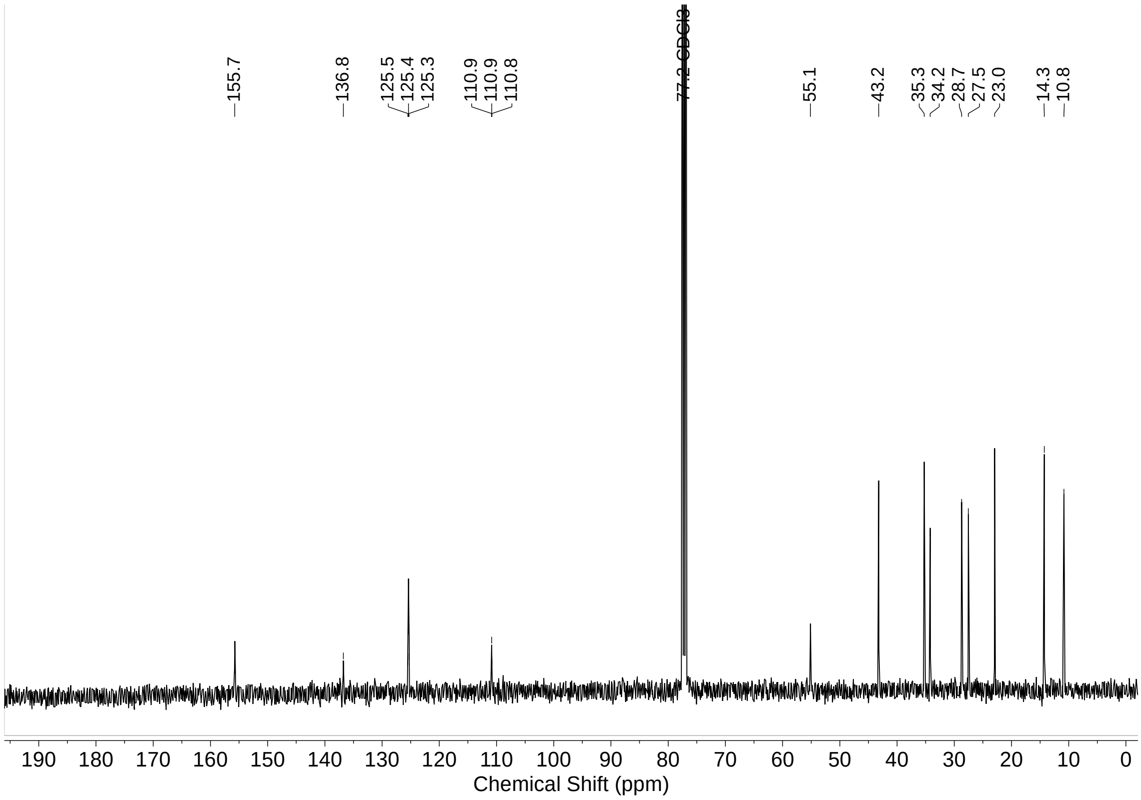

Figure S15: ^{13}C NMR spectrum of compound **3a** in $CDCl_3$.

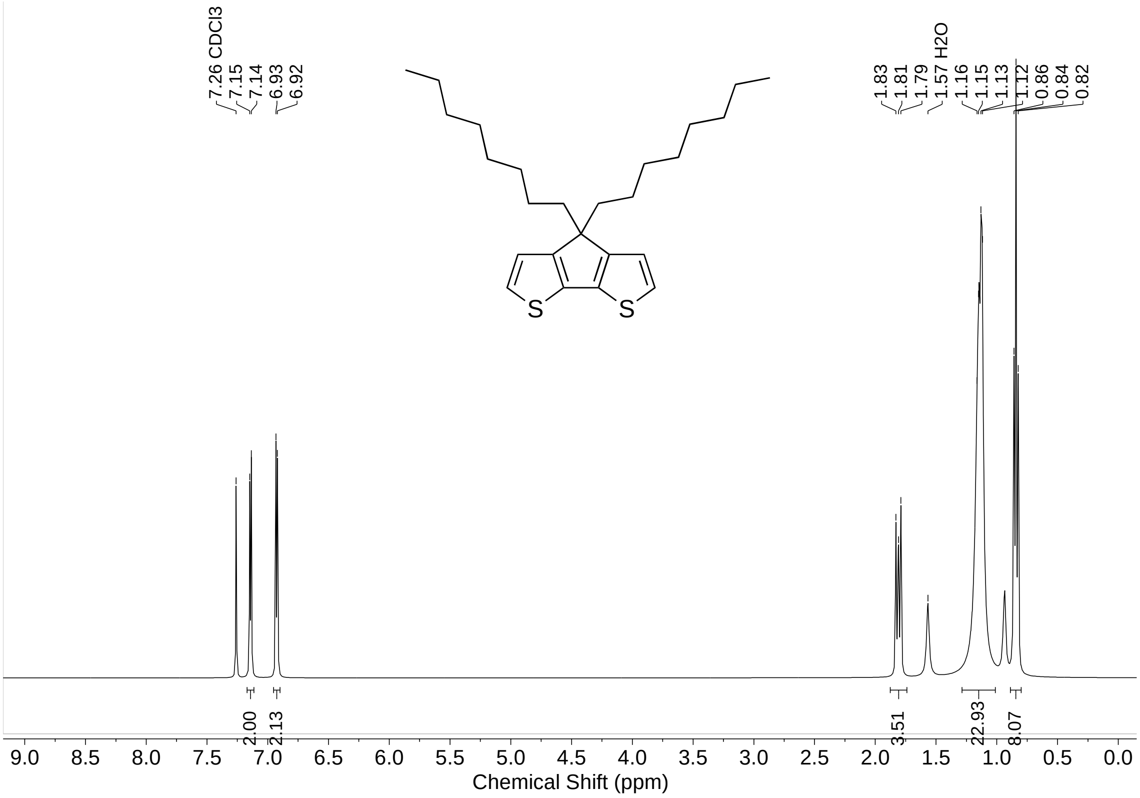

Figure S16: ^{1}H NMR spectrum of compound **2b** in $CDCl_3$.

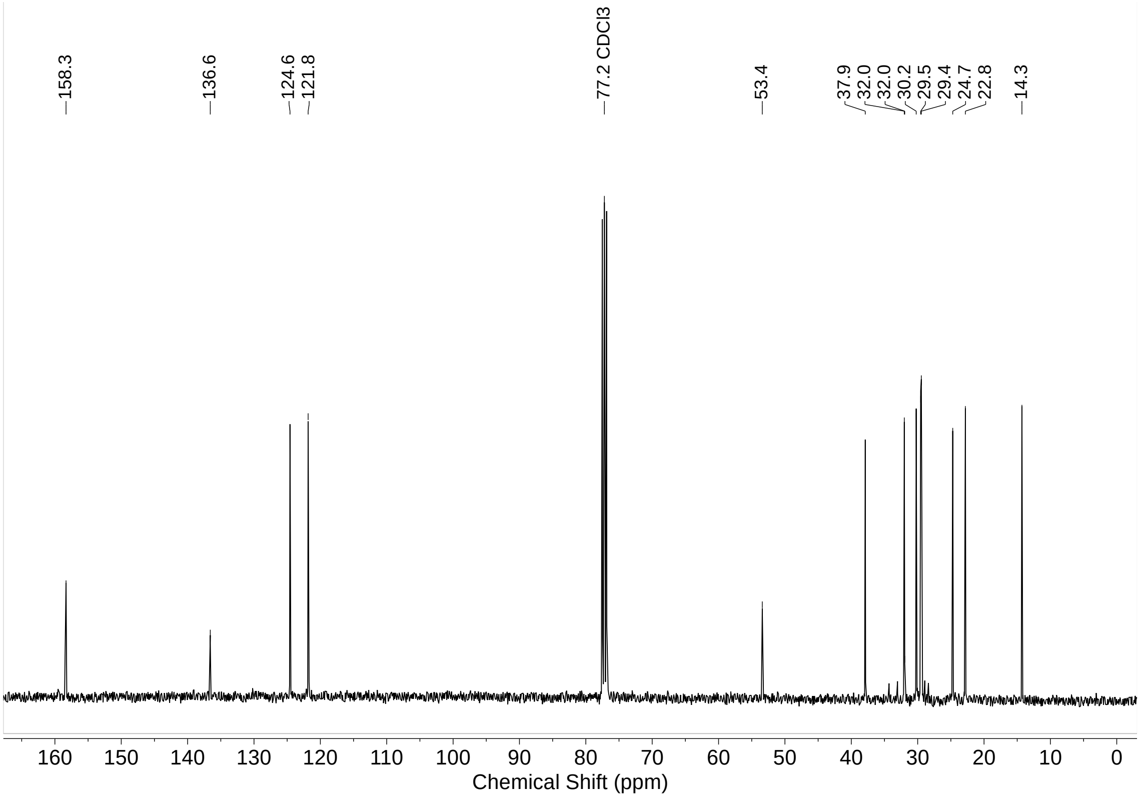

Figure S17: ^{13}C NMR spectrum of compound **2b** in $CDCl_3$.

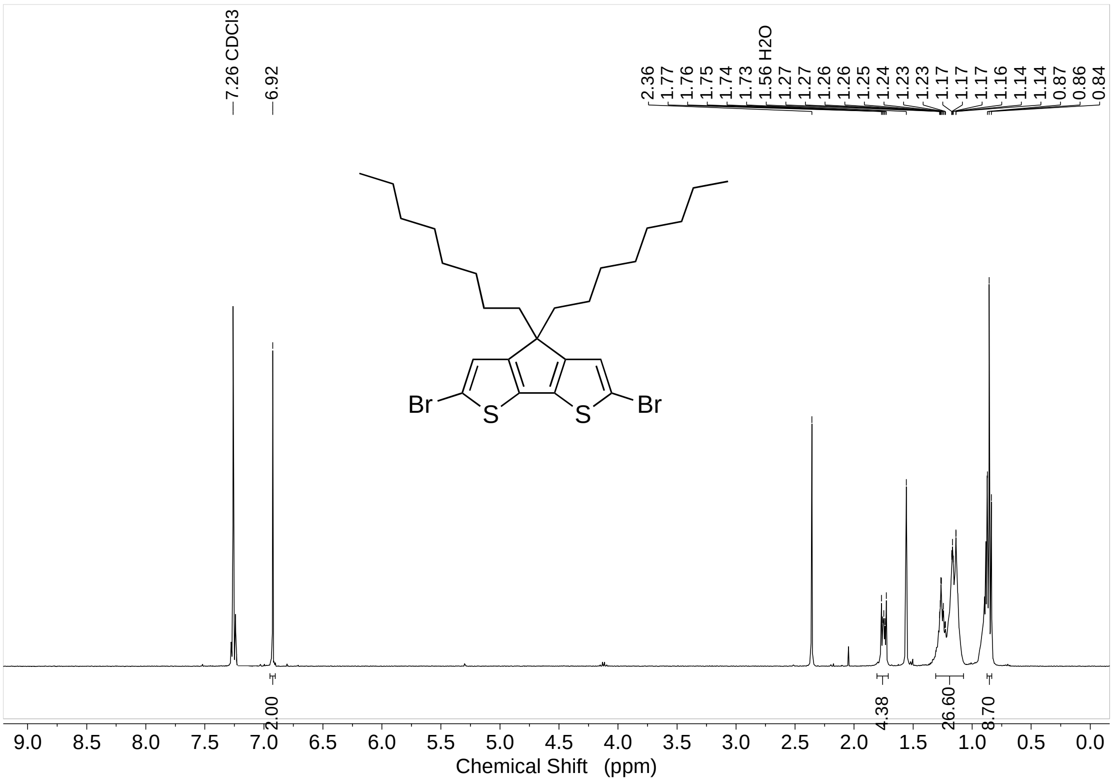

Figure S18: ^{1}H NMR spectrum of compound **3b** in $CDCl_3$.

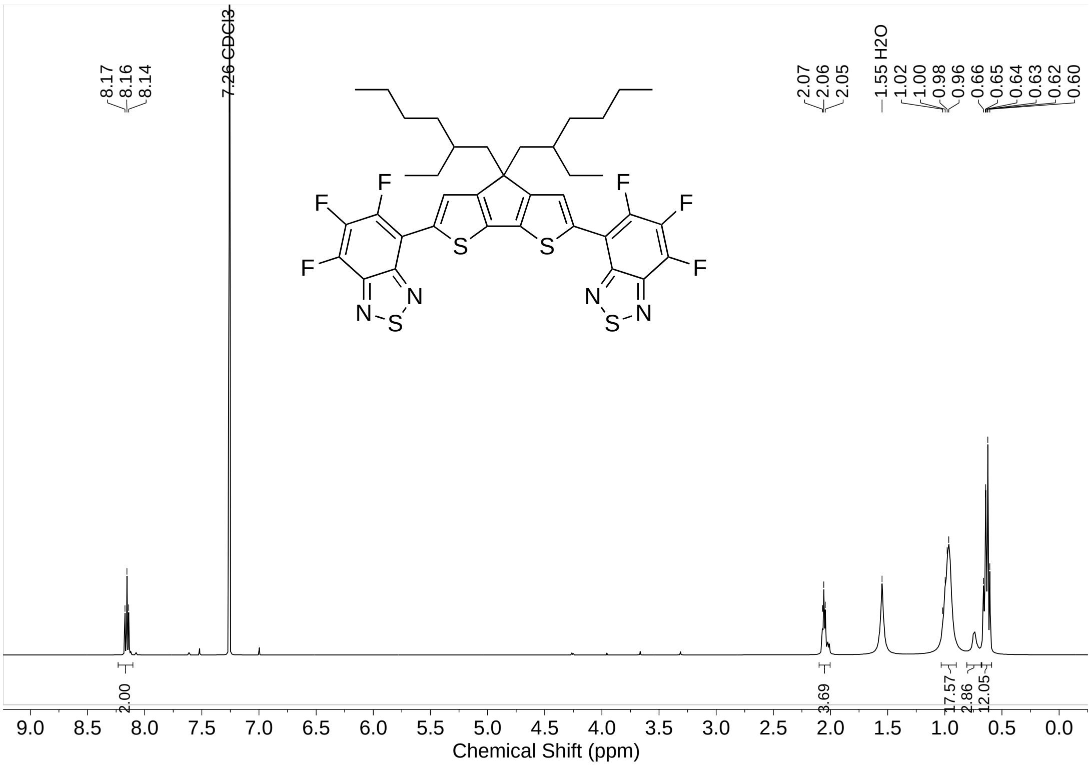

Figure S19: ^{1}H NMR spectrum of compound **EH-TFBT** in $CDCl_3$.

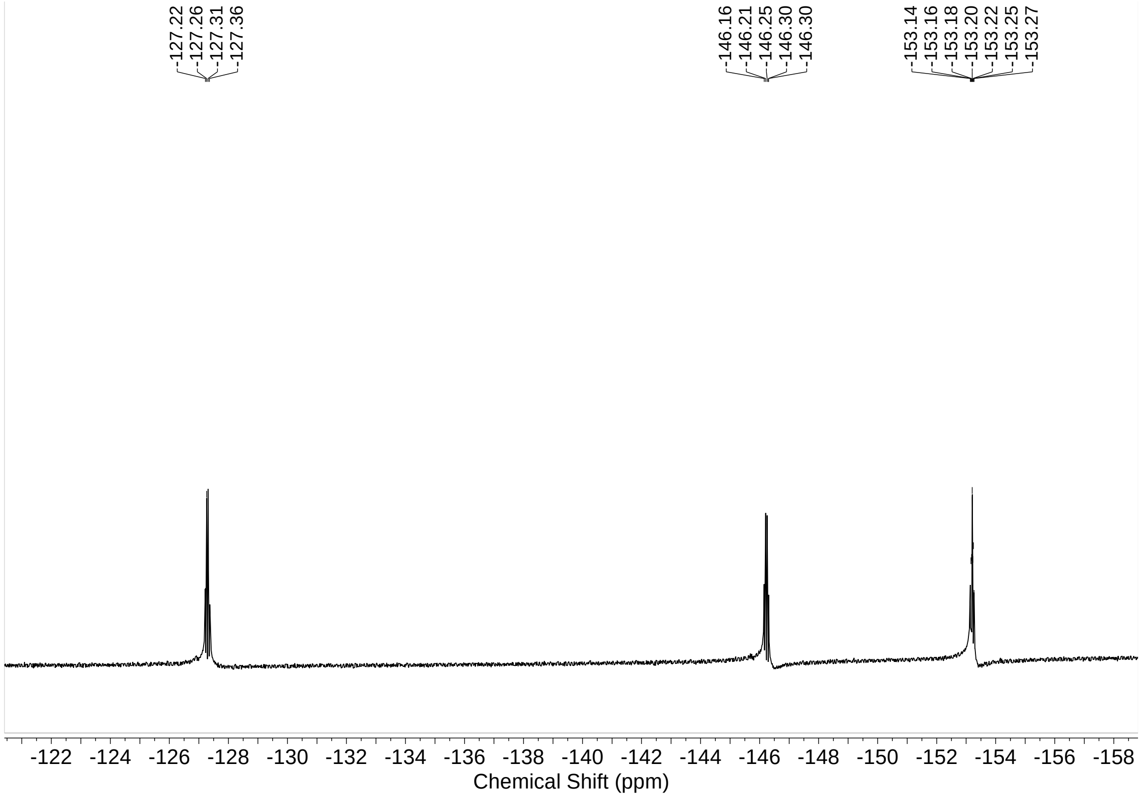

Figure S20: ^{19}F NMR spectrum of compound **EH-TFBT** in $CDCl_3$.

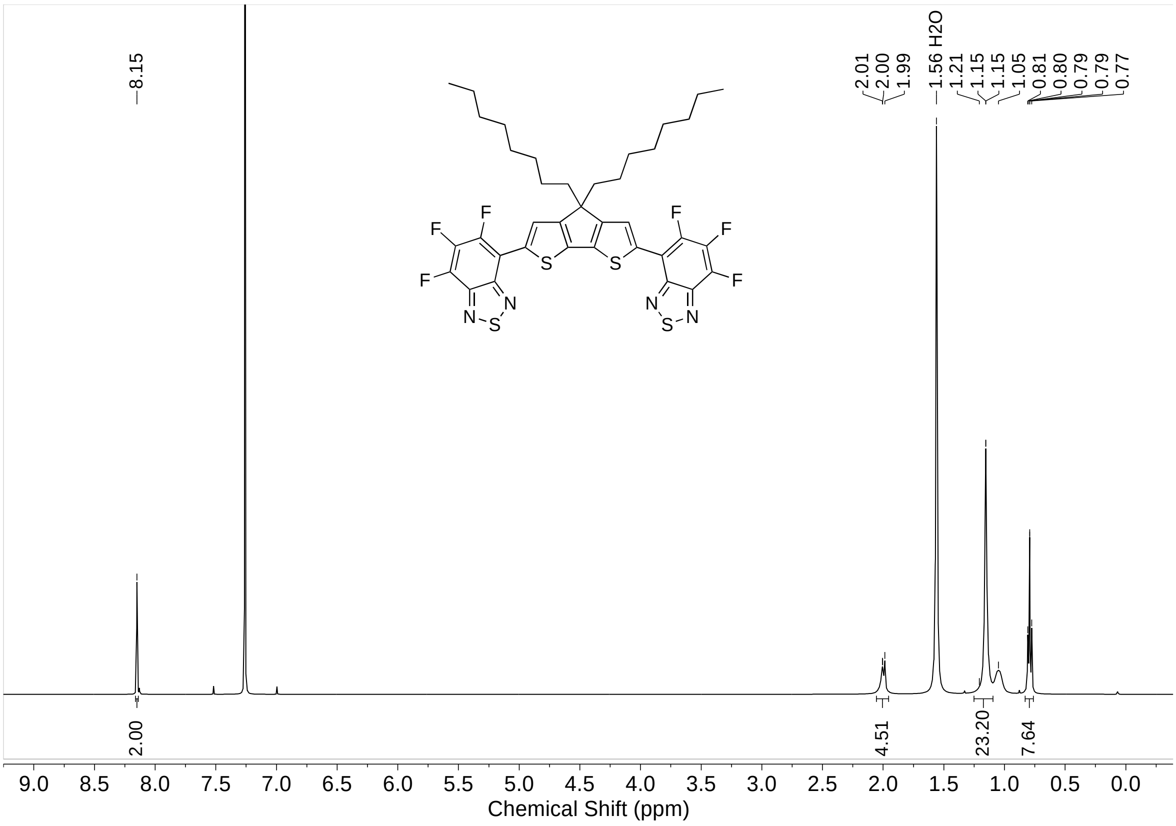

Figure S21: ^{1}H NMR spectrum of compound **O-TFBT** in $CDCl_3$.

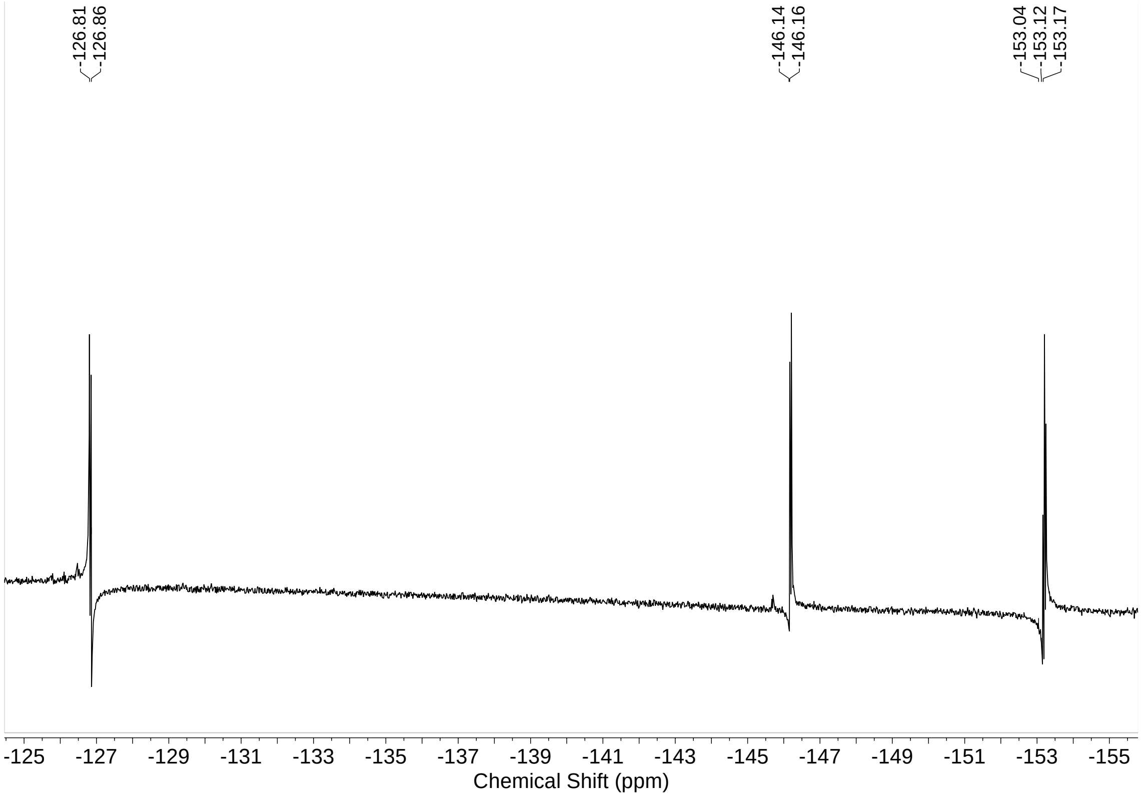

Figure S22: ^{19}F NMR spectrum of compound **O-TFBT** in $CDCl_3$.

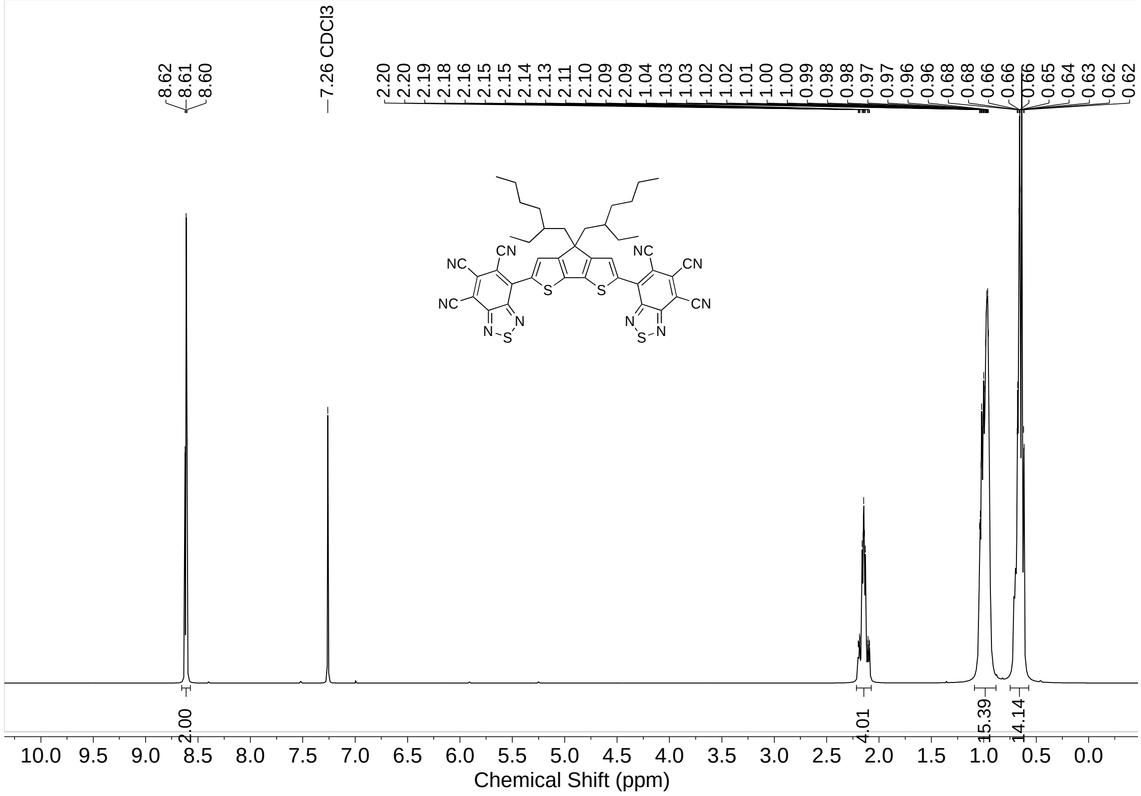

Figure S23: ^{1}H NMR spectrum of compound **EH-TCNBT** in $CDCl_3$.

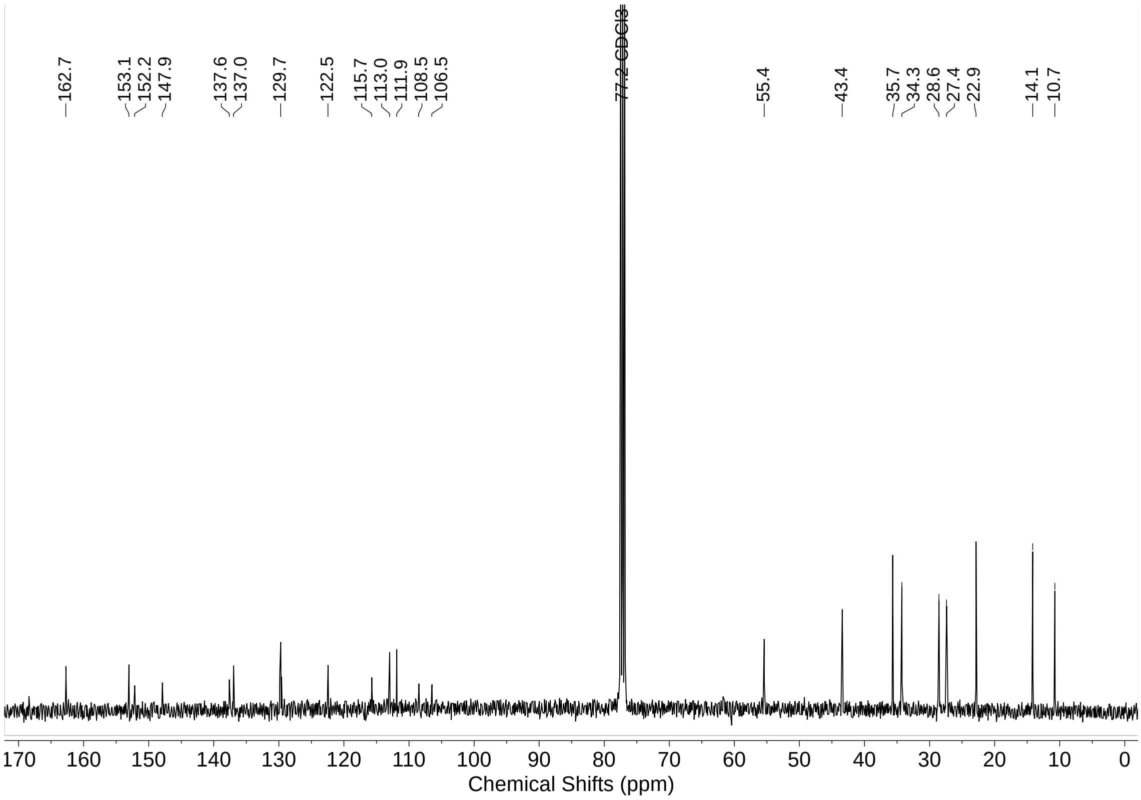

Figure S24: ^{13}C NMR spectrum of compound **EH-TCNBT** in $CDCl_3$.

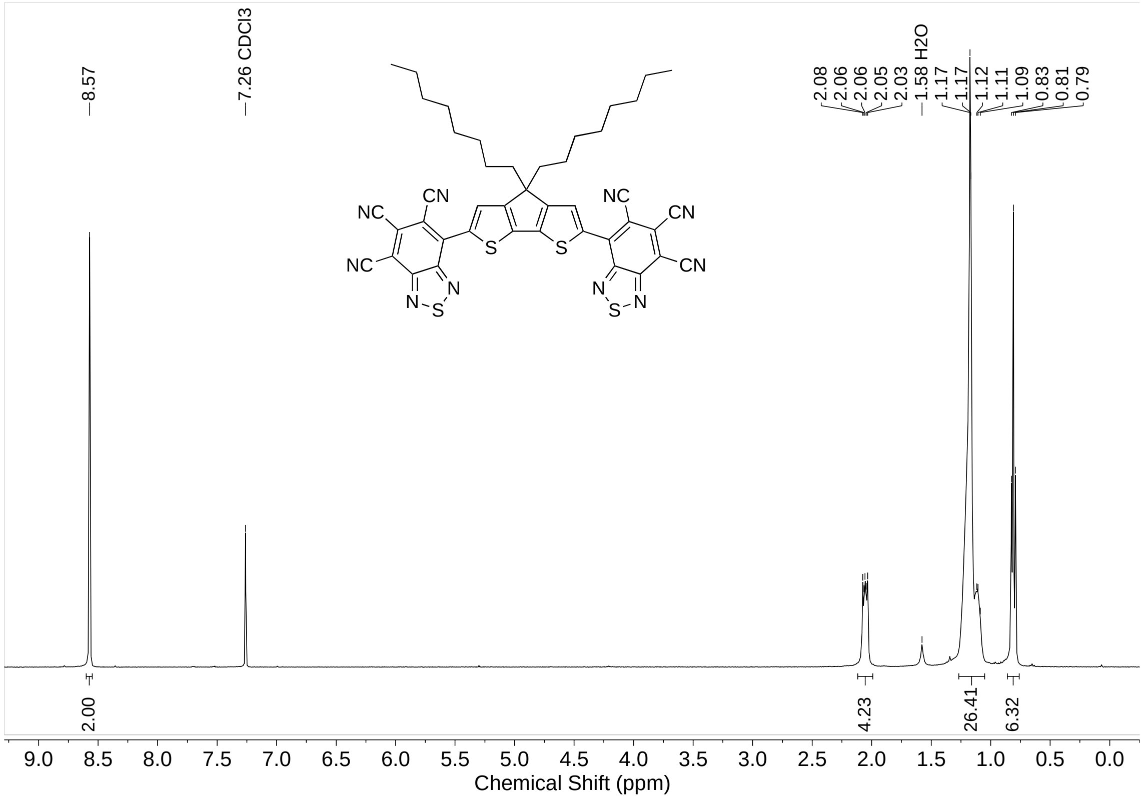

Figure S25: ^{1}H NMR spectrum of compound **O-TCNBT** in $CDCl_3$.

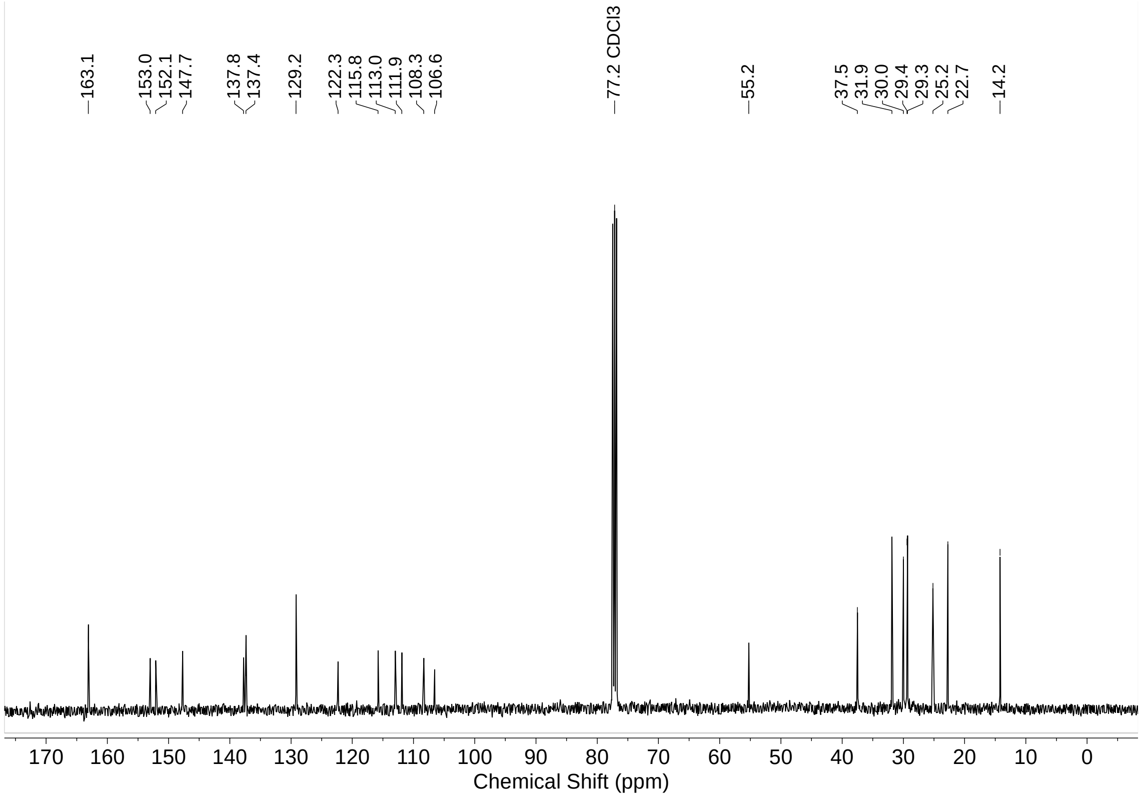

Figure S6: ^{13}C NMR spectrum of compound **O-TCNBT** in $CDCl_3$.

References

(1) Bard, A. J.; Faulkner, L. R. *Electrochemical Methods : Fundamentals and Applications*; John Wiley, 2001.

(2) *Gaussian 16 Rev. C.01*; Wallingford, CT, 2016.

(3) Becke, A. D. Density-Functional Thermochemistry. III. The Role of Exact Exchange. *J. Chem. Phys.***1993**, *98*, 5648-5652.

(4) Raju, T. B.; Gopikrishna, P.; Iyer, P. K. Highly Efficient and Facile Alkylation of 4H-Cyclopenta-[2,1-b:3,4-b′]dithiophene in Water. *RSC Advances* **2014**, *4*, 37738-37745,.

(5) Drozdov, F. V.; Surin, N. M.; Peregudova, S. M.; Trukhanov, V. A.; Dmitryakov, P. V.; Chvalun, S. N.; Parashchuk, D. Y.; Ponomarenko, S. A. Synthesis and Properties of Alternating Copolymers Based on 4H-Cyclopenta[2,1-b:3,4-b']dithiophene and 4H-Dithieno[3,2-b:2',3'-d]silol. *Polymer Science, Series B* **2019**, *61*, 56-76.
